# (Morpho)syntactic Variation in Agreement: Specificational Copular Clauses Across Germanic

**DOI:** 10.3389/fpsyg.2019.02994

**Published:** 2020-02-11

**Authors:** Jutta M. Hartmann, Caroline Heycock

**Affiliations:** ^1^General Linguistics, Faculty for Linguistics and Literary Science, Bielefeld University, Bielefeld, Germany; ^2^Linguistics & English Language, School of Philosophy, Psychology and Language Sciences, University of Edinburgh, Edinburgh, United Kingdom

**Keywords:** Germanic, copular clauses, agreement, downwards agree, number-only agreement

## Abstract

In this paper we bring together the results of our research into agreement in copular clauses in four different Germanic languages—Dutch, German, Faroese, and Icelandic—in order to provide an overview of the results. These cases present a particularly interesting window into how verbal agreement operates, since there are two potential controllers of agreement, which may disagree in person and/or number (*The source of the rumor* BE *the neighbors/you*-sg/*you*-pl). We will show that there is variation at all levels in which nominal controls agreement: cross-linguistic, inter-speaker within a single language, and intra-speaker. We argue that our data support the following claims: (1) “Downward” agreement for person, as well as number, with a nominal that is not in the canonical subject position is possible and in some cases preferred; (2) The agreement patterns observed in Icelandic and Faroese support the hypothesis that in these languages there are distinct Number and Person heads; (3) “Downward” agreement from a high position in the left-periphery is a grammatically distinct phenomenon from agreement when the verb remains in a lower position in the clause; (4) In some languages and some configurations, speakers show a significant degree of indeterminacy in their judgments and production, suggesting that speakers use more than one grammar. We relate our findings to current discussions in the generative literature on subject agreement and in particular differences between number and person agreement, and possible connections to restrictions on object clitics; we also discuss questions that remain open, and invite new, cross-disciplinary research.

## 1. Introduction

In this paper we bring together results from a series of experiments that we have conducted investigating agreement in a particular type of clause, across four Germanic languages: Dutch, German, Faroese, and Icelandic. Our investigation focusses on specificational copular clauses (SCCs henceforth), which feature minimally the copular verb (*be* in English) and two noun phrases (DPs). The definition of these clauses will be gone into in more detail below; (1) gives examples from English.


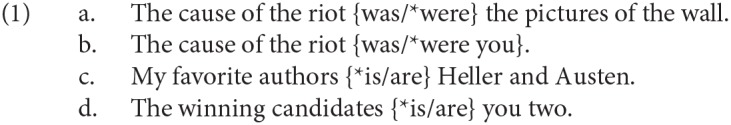


This type of clause is of interest for the syntax of agreement for various reasons. Notably, languages differ as to which of the two nominals the verb agrees with. As is suggested by the examples above, in English agreement is, to a high degree of consistency, with the leftmost/first DP (DP1); conversely, as discussed in Moro (1991, 1997), in Italian agreement is consistently with the rightmost/second DP (DP2):


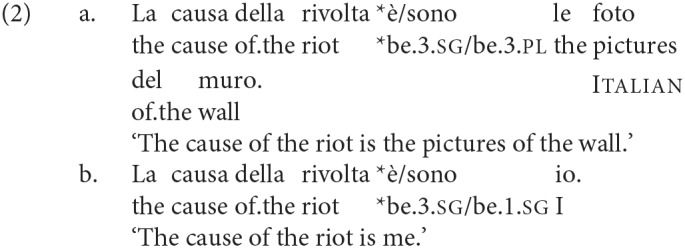


While there is general consensus in the literature that English and Italian are consistently “DP1 agreement” and “DP2 agreement” languages, respectively, in the syntax of these copular clauses, in this article we show that in other languages—even those closely related to English—there is a richer and more complex pattern of variation. We give an initial illustration in (3):


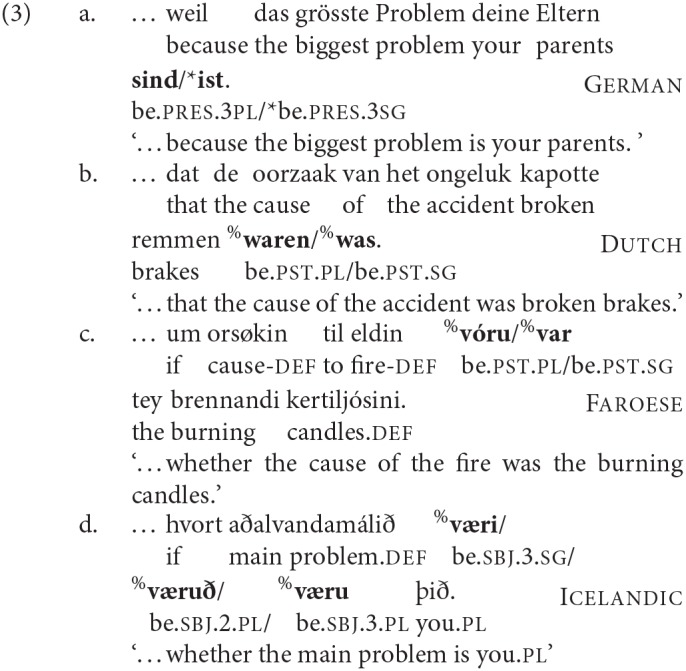


First, although in non-copular clauses all of these Germanic languages typically show a pattern very like English, in which the finite verb consistently agrees with a clause-initial subject^1^, here we find three different agreement patterns:

1. agreement in number and person with the precopular noun phrase (DP1 agreement), as in English;
2. agreement in number and person with the post-copular noun phrase (DP2 agreement), as in Italian;
3. agreement with the post-copular noun phrase in number only (number-only DP2 agreement)—see the Icelandic example in (3d).

Second, all of the four languages that we investigated allowed at least two of these patterns, but to different extents: Icelandic and to a lesser degree Faroese show all three patterns; Dutch only shows DP1 and DP2 agreement; and German almost categorically requires full DP2 agreement in all but one context. Third, all four languages—even German, which as just stated is almost categorical in the preference for DP2 agreement—show a notable shift toward DP1 agreement in one particular syntactic context, when the copula precedes both DPs, as in (4):


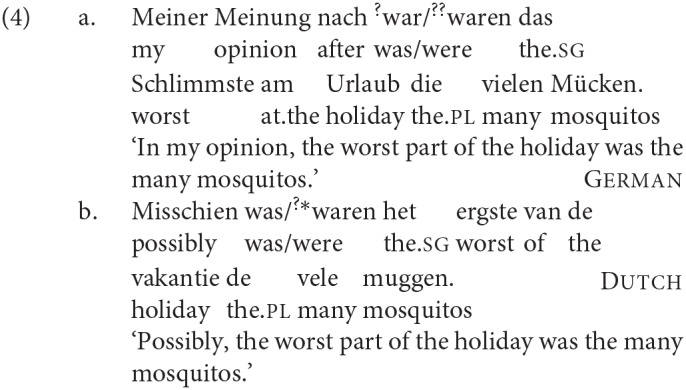


SCCs in these languages thus provide an interesting testbed for theories of agreement; in particular, for theories which predict severe restrictions on agreement with “low” nominative arguments, i.e., nominative arguments that appear in a position lower than the canonical subject position. They also present a new, relatively unstudied set of cases of agreement variability. In this paper we bring together the results from a series of experimental studies to give an overview of the generalizations that have emerged, and to relate these results to current theories of agreement. While the details of the goals and results of the individual experiments are available in a number of different papers, our aim here is to summarize the results, show the emerging overall picture and relate our findings to current issues discussed in the syntax of copular clauses and agreement. Our hope is that this will facilitate interdisciplinary discussion of the issues raised. Throughout, we will provide the references to papers where more detailed descriptions of experiments have been reported.

In section 2, we will outline some current issues in the syntax of agreement that are relevant to, and we hope illuminated by, our results. In section 3 we give some background on Specificational Copular Clauses and outline an argument that the agreement facts support an “inversion” analysis of these clauses. With this background, in section 4 we discuss patterns that are common to all four languages, and then in section 5 we turn to the variation we find, focussing in particular on person agreement. In section 6 we briefly discuss some of the new questions that have opened up in the course of this investigation, before concluding in section 7.

## 2. The Syntax of Agreement: Some Background

There is a range of theories on how the sharing of features that constitutes agreement can be modeled. In current generative grammar, it is generally assumed that morphological agreement is one possible reflex of a more general syntactic relation, Agree, that is established between a “probe” (the agreeing element, typically a head) and a “goal” (the agreement controller). There are a number of different proposals concerning the configurational relationship between the probe and the goal. (i) A longstanding position, going back at least to Chomsky (1981), but more recently championed in Koopman (2006), is that agreement holds between a head and an agreement controller in its specifier. (ii) A less constrained alternative is that Agree can be established between a probe and a c-commanding goal that may be more remote than the local specifier: see among others Wurmbrand (2012), Zeijlstra (2012), Bjorkman and Zeijlstra (2019). This is termed either “Upward Agree” or “Reverse Agree” because it reverses the hierarchical relations between probe and goal in the more widely adopted proposal of Chomsky (2000), namely that (iii) the probe must c-command the goal (“Downward Agree”). Depending on the framework and language considered, there is also work that argues for allowing both upwards and downward Agree (with upwards Agree often reducing to specifier-head agreement), see for example Béjar and Rezac (2003), Baker (2008), and Ackema and Neeleman (2018); note that for Béjar and Rezac (2003) the two types do not have equal status: upward Agree obtains only where downward Agree fails^2^.

In this paper we will be assuming downward Agree, for reasons that will become clearer when we have introduced the structure of copular clauses. (5) illustrates a simple case of how a downward Agree analysis handles subject-verb agreement in a non-copular sentence like *John has lived in Berlin*.


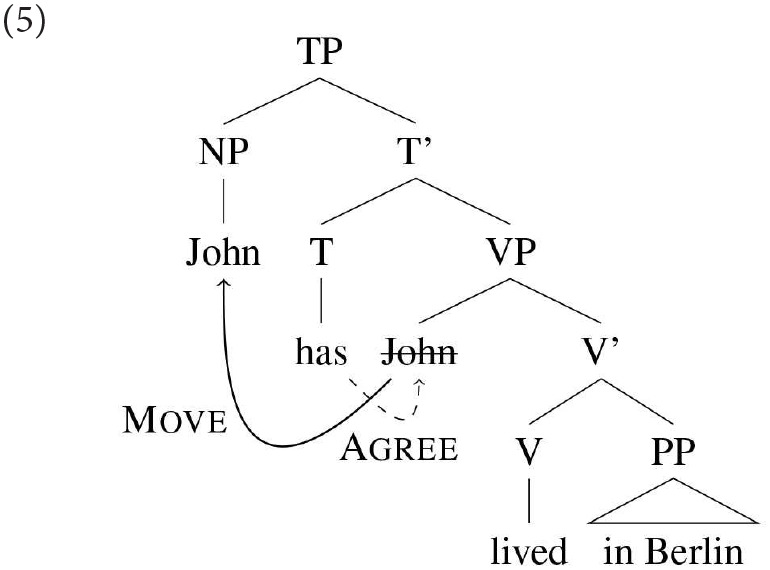


In this representation it is assumed that there is a single probe that has unvalued features for both person and number that will be valued by the first set of features that it encounters on a downward search of its c-command domain. Considerable work has been done on the idea that probes may be more or less specified in the features that they are searching for: e.g., a probe might be specified to match not against any person feature, but only, say, 1st or 2nd person, as proposed for Persian in Béjar and Kahnemuyipour (2017); again, we find cross-linguistic variation in this domain, see section 3.2 for further discussion.

There is now in fact a significant body of work establishing that agreement for person and agreement for number do not always behave in the same way; in some analyses it is argued that there are distinct syntactic probes, and in some cases in fact distinct heads associated with person and with number agreement. An argument for this last position is made in Sigurðsson and Holmberg (2008), based on “dative-nominative” configurations in Icelandic where there is a dative subject and a nominative argument lower in the structure. In such cases the verb may agree in number with a 3rd person nominative, as illustrated in (6a), but it cannot agree at all with a non-3rd person nominative, as illustrated in (6b)^3^.


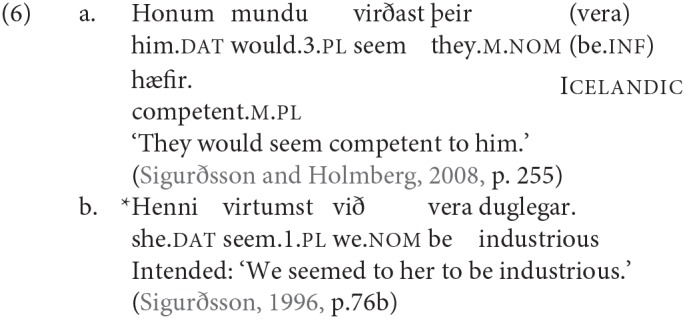


Differences between number and person agreement will be discussed in more detail in section 5.

Two requirements for a successful agreement relation to be established are thus that the probe and the goal must be in the appropriate hierarchical relation to each other, and that the goal must carry the features searched for by the probe. A third requirement is that there can be no “intervening” goal: Agree must establish a match with the first appropriate set of features in its search path (assuming downward Agree, this means that it will seek to match with the highest potential goal in its c-command domain).

In the case of morphological agreement, there also seems to be a further requirement: whether or not a DP with the relevant features can in fact control agreement depends on its morphological case. At least in the Germanic languages, there is a generalization that only nominative DPs can control agreement (see Bobaljik, 2008 for discussion, but also Jónsson, 2009; Ussery, 2017 for potential counterexamples). In most configurations, the nominative argument is the structurally highest argument, so in order to see the relevance of case, we need a configuration in which the two are separated. We find such a configuration in German with a number of psych verbs that select for a dative experiencer argument and a nominative theme argument. The dative argument has been shown to be the structurally higher argument (higher before any movement has taken place, and at the point that T is merged) with such verbs like *gefallen* in (7) (see Lenerz, 1977; Sternefeld, 2009, p. 563), subject-verb agreement is nevertheless with the nominative argument. This shows that nominative case is a precondition for agreement in German.


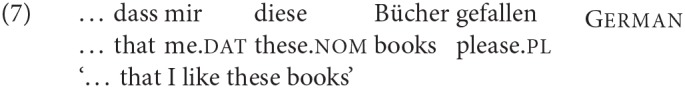


The Icelandic dative-nominative construction illustrated above in (6) shows a similar effect, but in these cases it has been argued that while the dative argument does not control agreement, it does interact with the agreement probe in some way (a phenomenon referred to in the literature as “defective intervention” see among many others, Holmberg and Hróarsdóttir, 2004; Sigurðsson and Holmberg, 2008; Thráinsson et al., 2015; Ussery, 2017; Hartmann and Heycock, 2018d); we will come back to this briefly in section 5.1.

In all the discussion so far we have been considering an agreement probe associated with finite T[ense], the goal of which is a nominative noun phrase (typically the subject), which results in morphological agreement on the finite verb. While this is the most familiar instance of agreement in Germanic, it has also been observed that in some Germanic languages, agreement with the subject of a clause can additionally be related to the C[omplementizer]-position. One version of this C-agreement is that there is agreement marking on the complementizer in a number of varieties of Dutch, as illustrated in (8), from van Koppen (2005), p. 33.


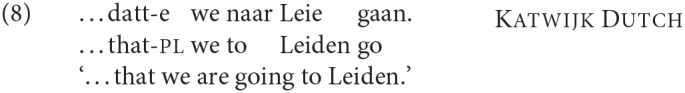


We will argue in section 4.3 that C-agreement is the basis for the agreement exemplified in (4b) above. But first we need to look also at the type of copular clauses that are the focus of our investigation.

## 3. Specificational Copular Clauses and Agreement

### 3.1. Specificational Copular Clauses: Background

Copular clauses may have various syntactic types of phrase in nonsubject position, including Adjective Phrases, as in (9a), Prepositional Phrases, as in (9b), among others.


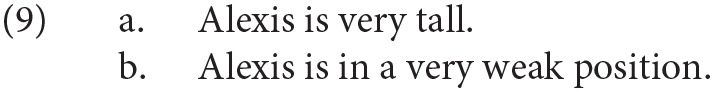


However, the case that is of interest to us here is that of “binominal” copular clauses, where both of the phrases that accompany the copular verb are nominals. Such binominal copular clauses have been further subclassified, the most influential classification being the four-way scheme set out in Higgins (1979) and illustrated in (10).


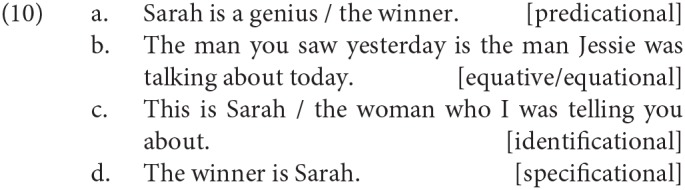


There is a substantial literature on copular clauses: for recent discussion and extensive references to other work, we refer the reader to den Dikken (2006b), Mikkelsen (2011), and Heycock (in press). Here we simply present a brief summary of some relevant distinctions from that literature.

The hallmark of predicational copular clauses like (10a) is that the pre-copular noun phrase, *Sarah* in (10a), is assigned the property described by the post-copular noun phrase, *a genius/the winner* in (10a). The post-copular noun phrase does not introduce a referent, even when it is definite (see Coppock and Beaver, 2015 for a recent discussion of the use of definite nominals as predicates). A syntactic diagnostic for predicative copular clauses in English that is often appealed to is that the same predication is felicitous in a small clause, without any instance of the copula:


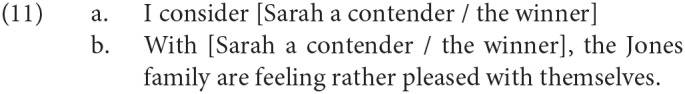


In equative/equational binominal sentences like (10b), two individuals are “equated”; put differently, the two descriptions are asserted to pick out the same referent. Such cases generally cannot appear in small clauses:


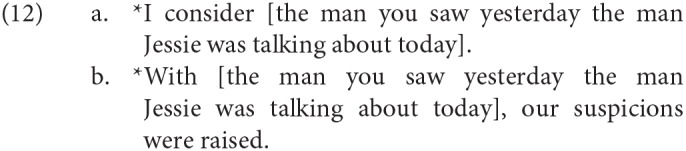


The ungrammaticality of examples like (12a), while frequently cited as following from the status of the small clause as an equative, is however already predicted by the fact that *consider* is a verb that requires its argument proposition to be open to subjective assessment (Saebø, 2009), and presumably being identical to another entity is not even coercible into a subjective predicate. Absolute adjuncts introduced by *with* are not subject to the same restriction, so that the ungrammaticality of (12b) does not suffer from the same confound^4^.

The third class, identificational copular clauses, illustrated in (10c), have a deictic expression as the first nominal and some referring expression (whether a name or a definite) as the second. There is some discussion in the literature as to whether such sentences should rather be subsumed into one of the other classes; see Partee (1986), Huber (2002), Mikkelsen (2005), Heller and Wolter (2008), and Moltmann (2013) for discussion.

The fourth class are specificational copular clauses (SCCs). This is the type that is our primary focus in this paper. One example was already given in (10d) above, some more are given in (13):


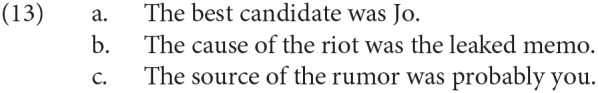


SCCs typically have a definite description as the first nominal, and some referring expression as the second. Since many definite nominals are ambiguous between a predicational and a referential reading, many sentences are ambiguous between a predicational and a specificational reading. Such sentences can give a sense of the kind of interpretation associated with “specification.” Consider for example (14):


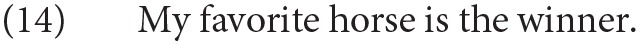


On the predicational reading, the sentence is a natural answer to the question *Has your favorite horse just won that race, or has it lost?* On the specificational reading, it is a natural answer to the question *Which horse do you like best, the one that won or the one that lost?* The predicational reading can be forced by adding a proper name as an apposition to the first DP:


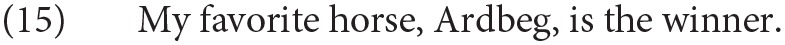


Equally, the specificational reading can be forced by a proper name in apposition to the second DP


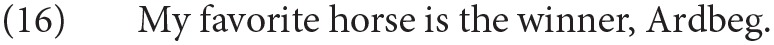


It is important to observe that in SCCs, at least in Germanic, the first DP occupies the canonical subject position, rather than some topic position high in the left periphery. Thus, for example, the subject of an SCC can immediately follow the auxiliary in a root polar interrogative in English:


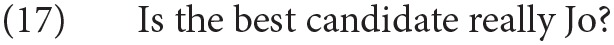


This distinguishes SCCs from cases that have been described in the literature as A′ predicate fronting, of the kind discussed in Birner (1992) and illustrated in the second sentence in (18) (the introductory sentence is included just to provide a favoring environment), where the same diagnostic indicates that the initial phrase does not occupy the canonical subject position. For extended discussion of the contrast between SCCs and A′ predicate fronting, see Heycock and Kroch (1998).


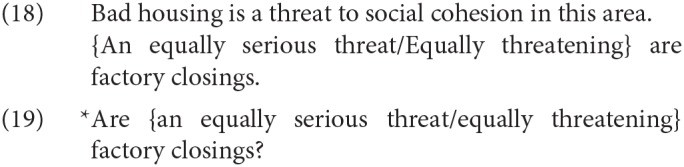


Although, as just discussed, the first DP in an SCC does not occupy some peripheral “topic” position, one of the best-known characteristics of SCCs is that they nevertheless have a fixed information structure. In particular, the second DP has to be in focus (Heggie, 1988). The following exemplification is from Heycock (1994). First, we see that the same predicative copular sentence can be used felicitously in both (20) and (21), where the questions set up either the first DP or the second as the focus in the answer:


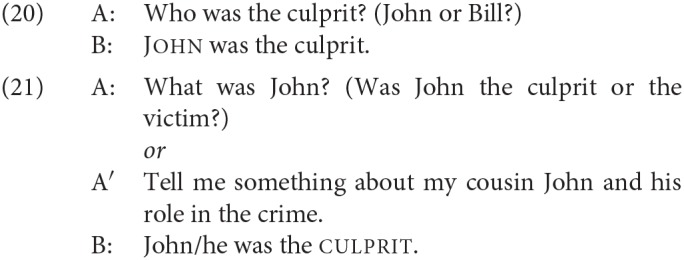


In contrast, the specificational sentence is good in only one of these two contexts, where the focus is on the postcopular constituent.


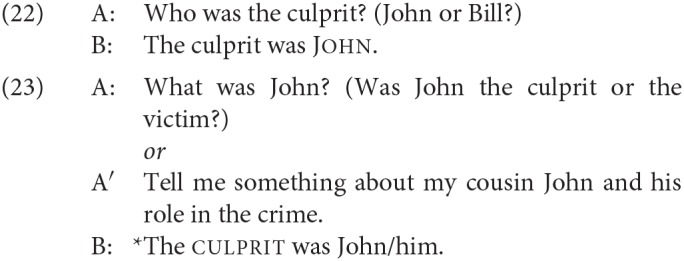


For experimental evidence of this restriction that makes use of the prosodic contours associated with focus, see Hartmann (2019).

A typical characterization of specificational sentences is that the first nominal, although in the canonical subject position, does not have a simple referential reading (of type *e*). In cases where this nominal could in principle pick out an animate entity, this can be seen by the pronoun used to refer back to it. Thus, while normally *the best candidate* would have to be referred back to by a gendered pronoun if it picks out a human candidate, this is not the case when it appears as the subject of a specificational copular sentence (Mikkelsen, 2005; Heycock, 2012):


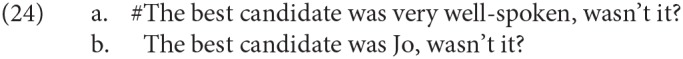


While there is general agreement in the literature that the second nominal in an SCC denotes an individual (in contrast to a predicational copular sentence), and that the first nominal does not, there is less consensus concerning the denotation of the first, and this relates closely to the analyses that different researchers have put forward. One widely-adopted proposal (see among others, Heggie, 1988; Moro, 1991, 1997; Mikkelsen, 2005; den Dikken, 2006a) is that the initial nominal is in fact a predicate (type <e,t>), and that specificational sentences are derived when the predicate, rather than the subject, of a small clause complement to the copula moves into the matrix subject position, as schematized in (25b). “F” is whatever functional head is taken to project the small clause; for most of the writers above it can be taken to be something akin to Bowers' (1993) Pr[edicative]P.


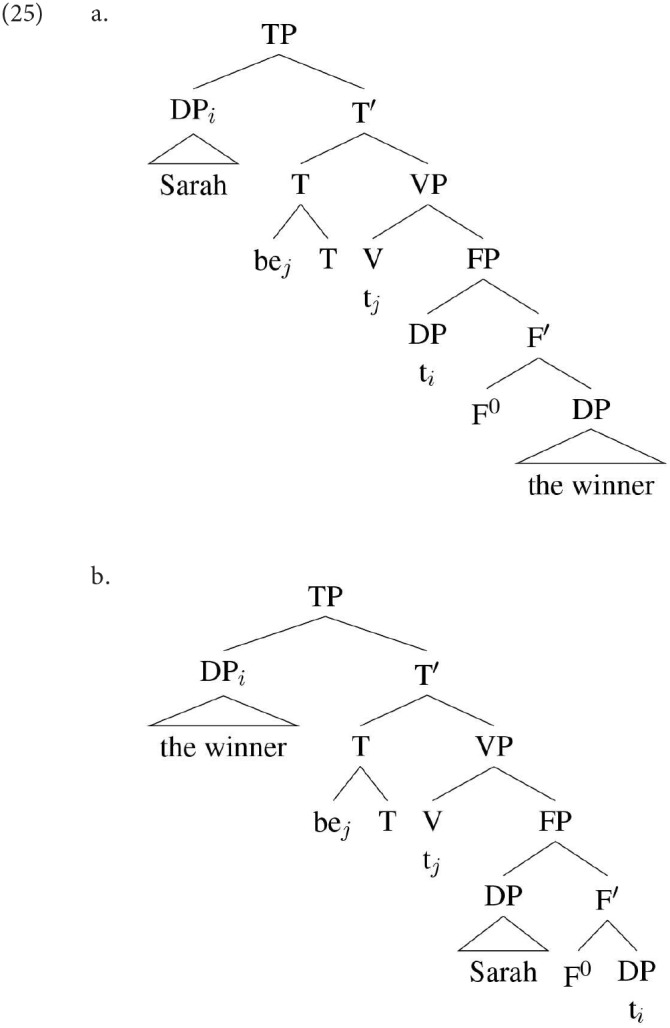


An alternative proposal for the interpretation of the initial nominal is that it is a concealed question, an interpretation available to definite descriptions in cases like (26) (Romero, 2005; Heycock, 2012):


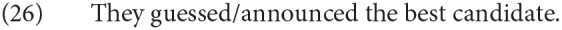


This proposal is still compatible with the syntactic “inversion” analysis schematized in (25), as a concealed question denotation can be shifted into a predicative interpretation (in the sense that it can combine with an argument of type e to yield a proposition) just like other definite descriptions, as discussed in Heycock (2012). The possibility of inversion will be important in the discussion to follow, but the precise nature of the semantic contribution of the first nominal will not be important here, so we will not be discussing it further.

Note that all inversion accounts have to explain why the higher DP within the small clause (DP2) does not “intervene” to block movement of the lower, preventing the inversion. There have been a number of proposals for how this problem could be circumvented. The essence of the proposal in den Dikken (2006a) is that the head of the small clause moves to adjoin to the copula *be*, and that this head-movement has the effect of making the two DPs within the small clause “equidistant” from a probe above. For Mikkelsen (2005), Shlonsky and Rizzi (2018), and Hartmann (2016) what is crucial is an informational asymmetry between the two DPs in a specificational sentence. As mentioned briefly above, specificational sentences are unusual in that they have a restricted type of information structure. We have followed the characterization of this as being a requirement that DP2 is in focus; this is the characterization that Shlonsky and Rizzi assume as well. An alternative characterization, adopted in Mikkelsen (2005), is that DP1 has to be a topic. Mikkelsen capitalizes on the informational asymmetry by proposing that the agreement probe on T may optionally carry a [+Topic] feature. If it does, and if in addition the lower of the two DPs (DP1) carries such a feature, then it may move past the higher DP (DP2), simply because that DP cannot match the probe. Shlonsky and Rizzi (2018), on the other hand, argue that DP2 in a specificational sentence moves to a low Focus position at the edge of the VP. In their terms, this is a “criterial position,” from which further movement is impossible (a case of “criterial freezing”). The remnant small clause may then move, stranding the focus to its right, and “smuggling” with it the lower DP, which subsequently moves out of it. In this paper we will assume that it is indeed the information structural asymmetry that is crucial in allowing the lower DP within the small clause to cross the higher, we will not discuss further the exact mechanism, but see Hartmann (2016, 2019) for a proposal.

There are a number of criteria that have been used as diagnostics for SCCs: as well as the distinctive pronominalization pattern for apparently animate nominals in initial position, illustrated in (24) above; these include restrictions on A′-extraction and obligatory focus on the second nominal (see Higgins, 1979; den Dikken, 2006b; Moro, 2006 for overviews and references). For the purposes of our studies, we operationalized the category of specificational copular clause as follows:


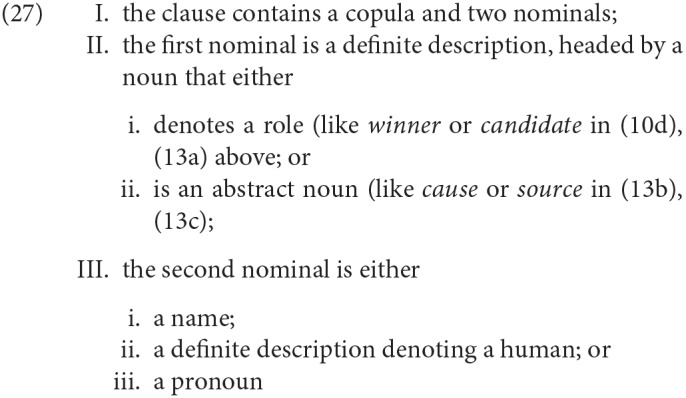


Clearly this operationalization would include some sentences that are at least ambiguous between specificational and predicational readings, as (14) above was. See section 3.3 for some detail concerning our strategies for avoiding such potential ambiguities in our materials.

### 3.2. Agreement in SCCs

The important work on specificational sentences in Moro (1991, 1997) showed that SCCs have different agreement properties in English and in Italian, as mentioned in the introduction. Essentially, in English agreement in SCCs is with the linearly leftmost/first/precopular overt nominal (DP1 henceforth), as illustrated in (1) above, repeated here as (28)^5^.


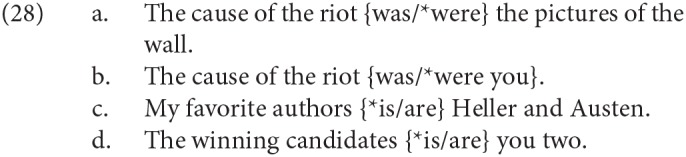


Note that, whether DP1s in specificational sentences are concealed questions or predicates, in either case they are predicted to be limited to 3rd person. They may be singular or plural, as just illustrated. In a specificational clause, if DP1 is singular, DP2 can be either singular or plural, and of any person. However, if DP1 is plural, the linearly rightmost/last/postcopular nominal (DP2) is again free to be of any person, but can typically only be plural, as in (28c,d)^6^. Evidently this restricts the types of potential agreement “mismatch” that can be constructed with this kind of specificational sentence. It should be noted, nevertheless, that the ungrammaticality of (28c,d) with singular agreement suggests strongly that the 3rd singular agreement in (28a,b) is controlled by DP1; if it were simply default agreement, the same 3rd singular agreement would be predicted to be acceptable in (28c,d), contrary to fact. We will return to this point in section 4.2.

The agreement pattern in Italian is different, as Moro argued: it is with DP2. This holds true both of number and person agreement. We repeat here the examples given earlier, which are adapted from those in Moro (1997), Ch. 1.


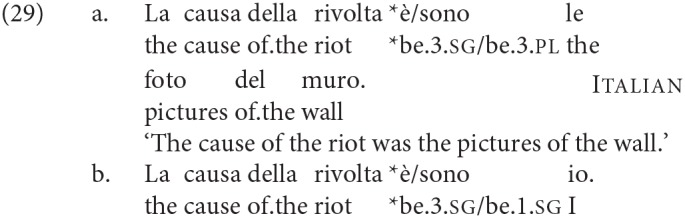


Moro (1997) derived this difference between English and Italian from the pro-drop character of the latter language. However, it has been known for some time that this cannot be the whole story. As pointed out in den Dikken (1998), Dutch allows DP2 agreement despite being a non-pro-drop language, and the same is true of German, which allows DP2 agreement even more freely (examples discussed for German go back at least as far as Berg, 1998).

If Dutch and German were invariant DP2 agreement languages (the characterization that Moro assumes for Italian), and English an invariant DP1 agreement language, one might pursue the idea that the difference in agreement is determined by the case properties of the languages. In Italian, Dutch, and German, both DPs in a finite specificational clause are nominative (in Dutch and Italian this is only evident when DP2 is a pronoun, since there is no overt morphological case marking on non-pronominal DPs in these languages). In Present Day English, on the other hand, where DP2 is a pronoun that is not syncretic for case, it is evident that it has to be accusative^7^:


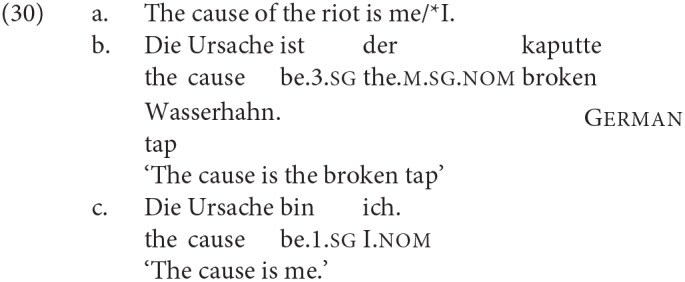


As discussed in the last section, in all the Germanic languages, only nominative DPs can control morphological agreement on the finite verb. Given that postcopular DPs in English specificational sentences are accusative (for whatever reason), this precludes the possibility of the verb agreeing with them. It might then be possible to set up a system that makes DP2 the first candidate for controlling agreement for some structural reason. In Italian, Dutch, and German the search for an agreement controller would stop there, yielding DP2 agreement; in English however, since DP2 agreement would be precluded by the accusative case, some mechanism could allow the search to continue, to find the nominative DP1 and agree with that.

However, Fischer (2003) established already that Dutch at least is not an “invariant DP2 agreement language.” Rather, there is significant inter- and intra-speaker variation between DP1 and DP2 agreement in this language even in SCCs, where DP2 is invariantly nominative. One of the goals of our work on agreement in SCCs in Germanic, then, has been to look in detail at four languages that all have nominative DP2 in SCCs: first to establish what the agreement patterns are in a number of configurations, and then to work toward an analysis that could explain the patterns observed. The languages that we chose to investigate are German, Dutch, Icelandic, and Faroese. All four are Verb Second (V2) languages, as will be discussed further below. German and Dutch have SOV order in subordinate clauses, while Icelandic and Faroese, like the other Scandinavian languages have SVO. All four languages show morphological agreement on the copula (unlike a number of other Germanic languages, including Afrikaans and the standard varieties of all the other Scandinavian languages), but German and Icelandic have “richer” (less syncretic) agreement morphology than Dutch and Faroese.

One possible line of analysis for the difference between DP1 and DP2 agreement in specificational sentences is developed in Béjar and Kahnemuyipour (2017, 2018). These authors adopt the kind of inversion analysis discussed above, according to which DP1 in a specificational sentence originates in the lower position within a small clause. However, rather than assuming that DP1 moves directly from this position to Spec,TP [as sketched in (25) above], they adopt the proposal that DP1 in a specificational sentence always moves initially to a position below T, which they take to be the locus of the agreement probe. They do not discuss the specifics of this position, but it seems that for the purpose of discussion we can identify it with Spec,vP:


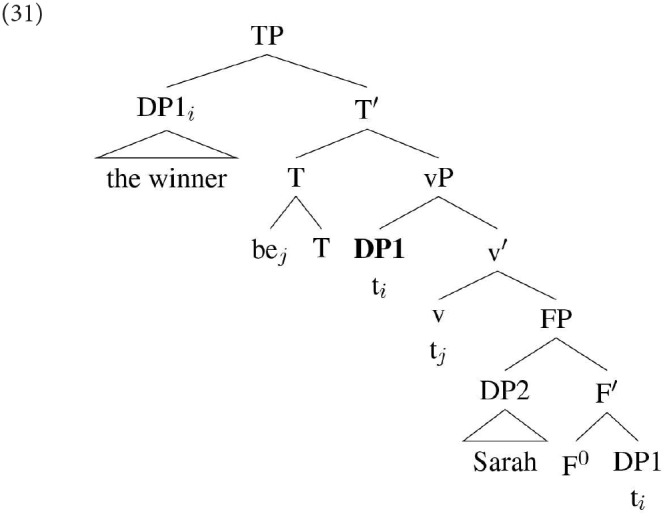


Given this derivation, DP1 in a specificational sentence, just like DP1 in a predicational sentence, will always be the first DP found by the agreement probe on T. The crucial extra assumption that Béjar and Kahnemuyipour (2017) make is that in a specificational sentence DP1 is deficient in ϕ-features. DP2 agreement then arises if a language has a probe that is searching for the feature(s) that DP1 lacks; such a probe will “skip” DP1 and hence be able to find and Agree with DP2. On the other hand, if a language has a sufficiently underspecified, and hence “undiscriminating” probe, it will match against DP1 and so agreement with DP2 will be blocked. In Béjar and Kahnemuyipour (2018) it is proposed that DP1 in a specificational sentence is deficient in that it lacks person features. Note that this lack of person cannot be common to all non-pronominal DPs, as it must distinguish between specificational subjects (which by hypothesis are skipped by a probe that is searching for person) and “ordinary” DPs occurring, say, as the subjects of predicational sentences (which are not skipped). Under this kind of analysis, variation between DP1 and DP2 agreement in a single language presumably reflects multiple options for the type of probe available (assuming, as is surely the case, that postulating variation in the ϕ-features of DP1 would be highly undesirable).

An alternative approach, which we have outlined in Hartmann and Heycock (2016, 2017) and follow-up work, is to propose that DP1 may fail to be agreed with in a specificational structure not because it is ϕ-deficient, but because in some languages it is possible for DP1 to reach a position above the agreement probe directly from its position within the small clause. Thus, while a derivation such as that in (31) will result in DP1 agreement, in some languages the derivation illustrated in (32) is possible:


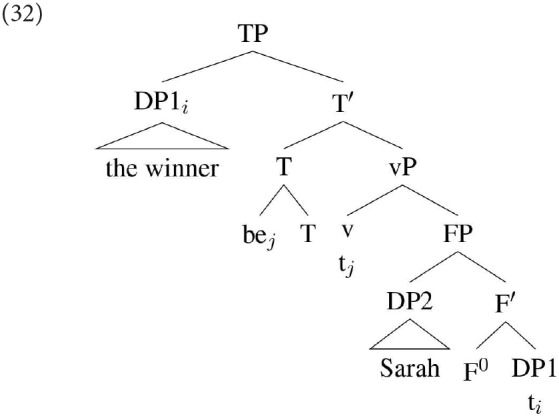


Assuming downward Agree, the highest DP within the c-command domain of T is DP2 (*Sarah)*: this then predicts that agreement will hold between T and DP2. In DP1's base position it will not be found by the agreement probe because DP2 is closer to that probe; and in its derived position it is above the probe, and hence not in a position for Downward Agree to reach it.

Under this view, rather than a difference in the ϕ-sensitivity of the probe, variation between DP1 and DP2 agreement reflects a difference in the initial landing site of DP1, possibly reflecting the presence of two distinct grammars.^8^ We will return to this issue after we have discussed how we have gone about trying to establish the essential facts about agreement in the languages we have been considering.

In order to begin to address the theoretical questions arising from agreement in these copular clauses, we have sought to address the following questions, which we will discuss in more detail in sections 4, 5.


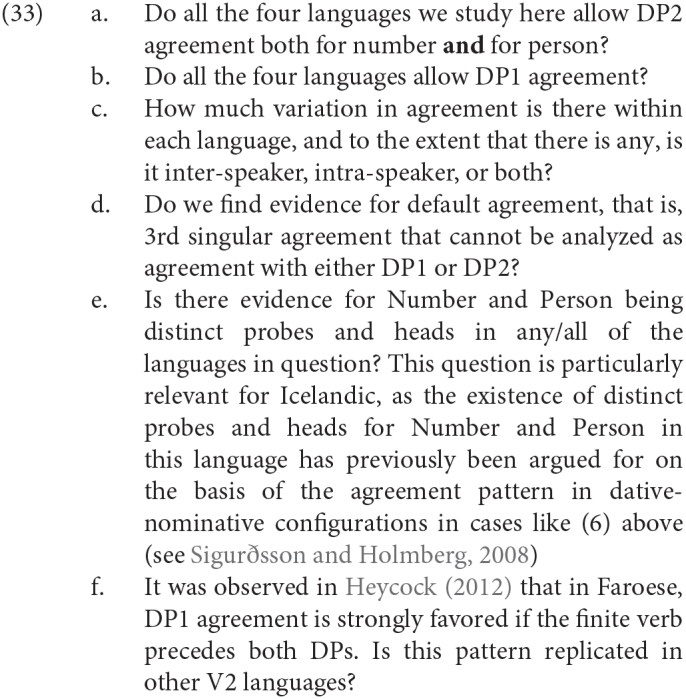


The questions in (33) aim at providing the overall picture of variation with respect to the availability of DP1 and DP2 agreement in different syntactic contexts. More specifically, potential differences in DP2 agreement with respect to number vs. person are interesting in the light of recent agreement theories, where it has been argued that downwards agreement with person is more restricted than number agreement, or—depending on whether or not we are dealing with a multiple agree configuration—possibly subject to syncretism effects. For recent analyses of agreement, the availability of DP1 agreement and the lack of default agreement is relevant for a distinction between a configurational approach to agreement in SCC (as we have proposed) and an approach such as Béjar and Kahnemuyipour (2017) in which DP2 agreement is claimed to arise just when DP1 does not have any ϕ-features accessible to the probe^9^.

Additionally, one important point of dispute in agreement theories is whether or not person and number should be taken to be different probes or in fact distinct heads in at least some of our languages, see (33e).

### 3.3. Methodological Issues and Strategies

As discussed above, while earlier work on agreement in SCCs assumed that each language was of a particular “type” (requiring either DP1 or DP2 agreement), more recent work on Dutch and Faroese (Fischer, 2003; Heycock, 2009) suggested that in at least some cases there is intra-language variation of various kinds. Some of this variation is conditioned by syntactic environment (see in particular section 4.3), but some is not (or at least, not evidently). Given the possibility of inter-speaker variation, in order to understand the status of the different agreement options in a language it is essential not to rely on data from a single consultant.

In order to investigate the available patterns of number and person agreement with two nominative DPs in SCCs, we therefore conducted several experimental studies on number and person agreement in Dutch, Faroese, German and Icelandic, combining production studies (fill-in-the-blank) and rating studies (thermometer rating, following Featherston, 2008, which is a variant of the magnitude estimation technique, see Bard et al., 1996). We chose to investigate the issue using both production and rating tasks, as both have their advantages and disadvantages. A production task, such as the fill-in-the-blanks paradigm that we used, allows but does not force speakers to reflect on their own production. This method has been used in previous studies (see Berg, 1998; Fischer, 2003; Heycock, 2009). A further benefit of such a production task is that it allows for speakers to produce forms that the investigators were not previously aware of. However, the production task is to some extent a forced-choice task, in that participants are presented with a sentence in a particular order and can only choose some form to fit a single blank. Hence for example a 50/50 distribution might reflect two fully acceptable options (potentially, completely free variation between two grammatical variants) or two equally degraded options. The rating task can reveal such distinctions.

It has to be acknowledged that these experiments can only be viewed as a preliminary exploration, as there has been no prior work on this topic on Faroese and Icelandic, and little on Dutch or German. In particular, our experiments were not designed to easily reveal the extent of intra-speaker variability, since each participant saw at most 3 examples of each condition. We have made some preliminary attempts to look at individual speakers, and to establish to what extent it is possible to identify dialect splits: for this we refer the reader to Hartmann and Heycock (2017, 2018a). Further, for logistic reasons we had to conduct most of our experiments on-line, so that they had to be of limited length, and it was not possible for us to have the same participants do both the production and rating tasks. As pointed out by a referee, this is worth bearing in mind in the context of discrepancies that we found in some cases between production and rating data, discussed below.

The experiments were designed and run as parallel as possible to allow for cross-linguistic comparison of the overall patterns/effects, even though direct comparison of individual ratings and productions is not possible^10^. All studies were set-up online using the OnExp online software package^11^. Test sentences and fillers were presented one per screen in randomized orders per participants.

Participants were recruited via personal contacts for Faroese, Dutch, and Icelandic and we additionally used the mailing list “Onze Taal” for Dutch. Experiments for these three languages were run fully online. Participants could sign up to take part in a lottery for a gift voucher after having finished the online study. The studies on German were all run on-site at the University of Tübingen, with individual payment for participation. All participants had to state their mother tongue(s), we only included the data of the participants who declared themselves to be native speakers of the language we were investigating (none of the participants reported themselves as bilinguals). As the test sentences were distributed across various lists, we analyzed the data with roughly an equal number per list per experiment. Per study we had between 8 and 15 participants per list, which adds up to between 50 and 90 participants per study.

For the production studies, participants were presented with sentences with a blank in one position of the sentence, as in (34), which participants were asked to fill with a single word of their own choosing.


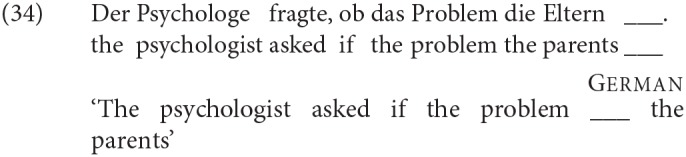


Before the actual study started, participants went through a short practice phase. All studies included fillers, between 1.5 and 2 times as many as the actual test sentences.

In analysing the data, we excluded all cases where participants used a verb other than the copula. All included cases were coded for number and person agreement on the copula, and then as DP1 and DP2 agreement (plus number-only DP2 agreement in Icelandic, see below). For the statistical analyses, we calculated relative frequency of DP1 agreement. These values (f) were transformed as usual, i.e., arcsine(square-root(f))—and we calculated planned contrasts with participant (F1) or item (F2) as random factors. Where appropriate, we also looked at the variation within and between speakers in more detail.

The rating studies followed the Thermometer Rating task model described in Featherston (2005), a variant of the Magnitude Estimation technique (Bard et al., 1996). Participants are asked to rate the naturalness of a sentence in relation to two reference sentences. The reference sentences are provided with a fixed score: one, a rather natural sentence, is assigned the value 30, one, a less natural sentence, is assigned the value 20. Reference sentences were kept on the screen throughout the experiment; stimulus clauses were presented one at a time, with participants advancing to the next by button press, with no possibility to return to earlier screens. Participants were asked to rate the naturalness of individual examples by providing numerical scores (all positive numbers) for individual sentences. As with the Magnitude Estimation technique, this allows participants to make finer grained judgments and to make distinctions between more or less unacceptable sentences. Before presenting the study, participants went through two short practice phases: the first one gave participants practice in assigning a value to the length of a line in reference to two standard lines assigned the values 30 and 20. Then they practiced rating naturalness with a set of sentences that varied in naturalness, so that they could familiarize themselves with the task.

The resulting scores for the rating experiments were all z-transformed (including fillers) per participant in order to normalize for the different scales participants might still have used. Z-scores were aggregated within conditions for each participant (F1) or item (F2). Where possible and useful, we computed the difference between DP1 and DP2 agreement for participants or items by subtracting DP1 z-scores from DP2 z-scores (positive values indicate that DP2 agreement is overall rated higher; negative values indicate that DP1 agreement is overall rated higher). This procedure allows us to investigate the same contrasts for the production and the rating studies. Depending on the design and more specific goals of each study, we also analyzed the rating data independently from the production data using ANOVA and mixed effect models (see the respective papers for details), especially when considering potential correlations with other factors, or speaker-groups.

All rating studies included a range of different filler sentences, including a set of standard-setting sentences, which help to put the overall acceptability into perspective (along the lines of the ideas presented in Gerbrich et al., 2019).

To test for number agreement with either DP1 or DP2 we used singular DP1 and plural DP2, corresponding to (35a)^12^; to test for person agreement we used singular DP1 and non-3rd person DP2, corresponding to the English example in (35b) (note that in all the Germanic languages that we investigated other than English, the pronouns for 2nd person singular and plural are distinct):


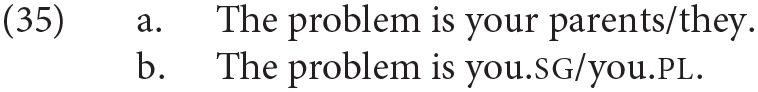


While keeping the studies in the different languages as similar as possible, we needed to make adjustments in the design of the experiments due to language specific differences in the morphology of the copula; e.g., Dutch and Faroese do not make person distinctions in the plural, while Icelandic and German do; Icelandic and German on the other hand have syncretic forms for 1/3sg (in both present and past tenses in Icelandic, only in the past tense in German). Additionally, the pronominal forms in Dutch and German have a syncretic form for 3sg feminine and 3pl (*zij* and *sie* in Dutch and German, respectively). In order to avoid ambiguity in production and rating, where we needed a 3rd person plural we could not use a pronoun in these languages but rather had to use a nonpronominal DP.

It was mentioned earlier that individual examples of binominal copular sentences—particularly taken out of context—may be ambiguous, or simply indeterminate. We note here the principal ways we sought to avoid this in our materials:

- In the experiments reported here, DP1 was usually headed by a singular non-animate abstract noun [option II.ii in (27) above] like *reason/cause, problem, hope, inspiration* etc.: e.g., *The reason for the delay* *be* *their friends*, except when DP1 needed to be plural (which is not possible with all of these abstract nouns). In the right context it is certainly possible for speakers to use even such abstract nouns to refer to individuals in a kind of metonymy (e.g., *The reason for the delay just walked into the room*), so in principle a copular clause like *The reason for the delay is my husband* is ambiguous between a specificational reading (≈ My husband caused the delay) and a predicational one (≈ The reason for the delay is related to me by marriage), but to our ears the predicational reading is much less readily available.
- Additionally, we did carry out experiments that included conditions where DP1 was headed by a noun denoting some kind of role [the option described in II.i in (27) above], like *the most likely winner(s), the only witness(es), her favorite drinking companion(s)*. This is the type of DP1 that seems most likely to create ambiguities, as DPs like *the winner* can more easily be used to refer to individuals than DPs like *the problem*. This “role” type of DP1 was used almost exclusively when we wanted to test the possibilities for agreement when DP1 is plural, since they are more natural in the plural than the DPs headed by many abstract nouns. For example, we take (36b) to be more natural than (37b):
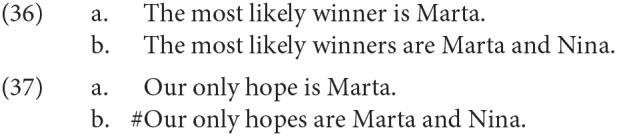
  In most cases where we used these “role” type DP1s, however, DP2 was a pronoun (and hence unlikely to be given a predicative interpretation, as mentioned above). Further, in our production experiments in Icelandic and German we did a direct comparison between a condition where DP1 was headed by a “role” type noun (e.g., *The most likely winner ___ you*.*sg*), and a condition where DP1 was headed by an abstract noun (e.g., *The main problem ___ you*.*sg*), and we found no difference in participants' choice of agreement on the copula in the two conditions (Hartmann and Heycock, 2017, pp. 249–261).
- In all conditions where we were testing for the distribution/acceptability of **person** agreement in SCCs, DP2 was a pronoun (*The reason for the delay* *be* *you*). 1st and 2nd person pronouns have a predicative use only in very restricted circumstances (see e.g.,Percus and Sharvit, 2014), so all such cases are highly unlikely to get a predicational construal.
- When we were testing for the distribution/acceptability of **number** agreement in SCCs, DP1 was always singular and DP2 plural. This also strongly disfavors a predicational reading (and, clearly, also an equative one). Consider the examples in (38):
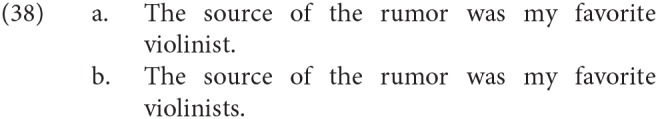
  (38a), where both DPs are singular, can with some considerable effort get a predicational interpretation, where “the source of the rumor” is taken to be a very indirect way to describe an individual (*the source of the rumor—my friend Michael—was my favorite violinist*). But (38b) clearly cannot get such an interpretation: its only interpretation is as an SCC.

All four languages that we discuss are Verb Second (V2) in root clauses: thus in a root clause, the initial position is not reserved for subjects. This creates possible confounds that do not arise in English, where topicalization of a predicative NP is not string-identical to the specificational order, as show in (39)^13^:


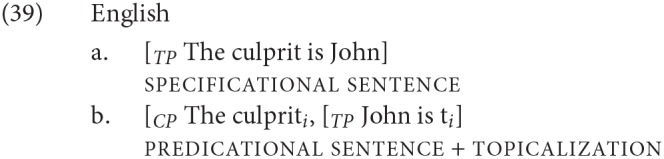


We discuss in section 4.1 how we avoided this confound.

The production and rating studies on number included root clauses as well as embedded clauses in order to evaluate the possible effect of embedding (that is, of non-V2 vs. V2 structures), the experiments considering person only included embedded contexts in Icelandic, Faroese, and Dutch. In the German production study for person, we tested root clauses.

Based on our work on production of number agreement in Faroese and Icelandic (Heycock, 2009, 2012; Hartmann and Heycock, 2016, 2017), rating of person agreement in Icelandic (Hartmann and Heycock, 2018d), rating and production of person agreement in Faroese (Hartmann and Heycock, 2018e), rating and production of number agreement in Dutch and German (Hartmann and Heycock, 2018b) and rating and production of person agreement in Dutch (Hartmann and Heycock, 2019), we have arrived at the following answers to the questions raised in (33):


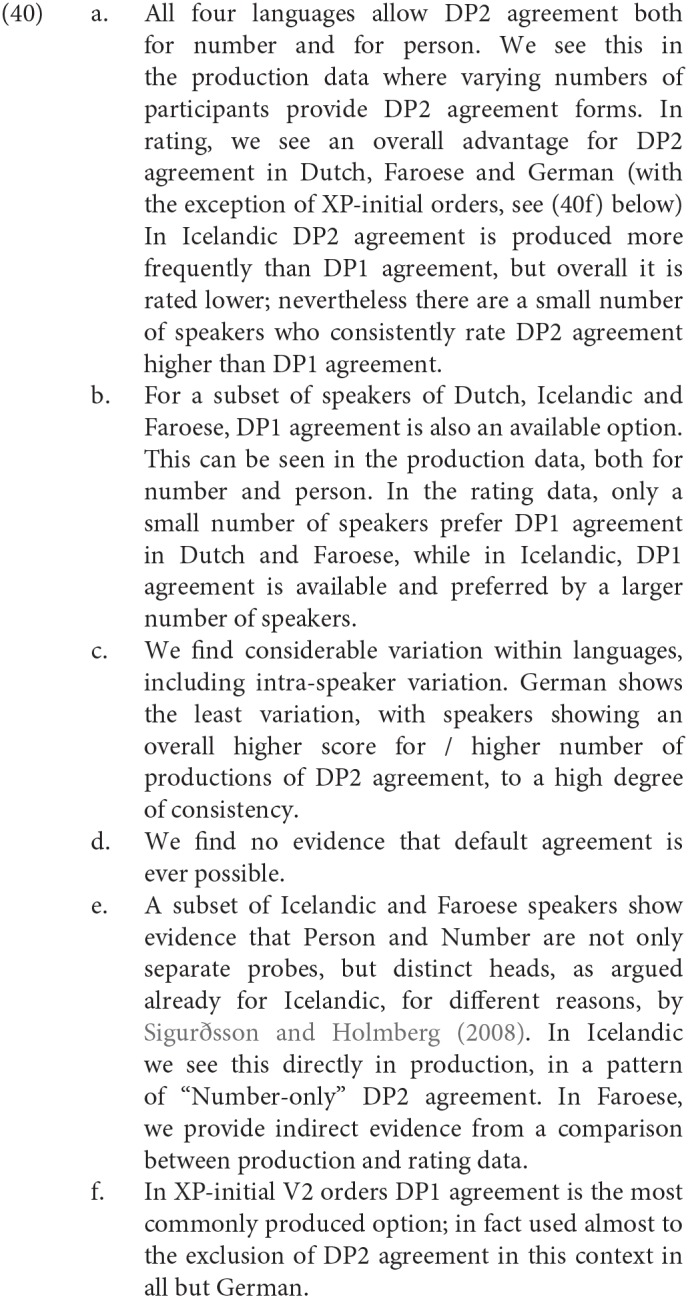


We will discuss the patterns (40a), (40d), and (40f) that all four languages share in section 4, and the other patterns, which we find only in a subset of the four languages, in section 5.

## 4. Shared Patterns of Agreement Across Dutch, Faroese, German, Icelandic

Our investigations of copular clauses in Dutch, Faroese, German and Icelandic show that all four languages share two patterns of agreement. First, all four languages allow agreement with DP2 both in V2 clauses, but also—more significantly, given the issues of ambiguity in V2 structures outlined in the last section—in non-V2 contexts. None of the languages we investigated show evidence for default agreement. Additionally, we find that all four languages show high levels of use of DP1 agreement in adjunct/modifier-initial V2 structures (that is, root clauses that have the order XP–*be*–DP1–DP2). We discuss the three patterns in the following subsections.

### 4.1. DP2 Agreement

In all four languages we found a high level of production of DP2 agreement in root (V2) clauses. When DP1 was 3rd singular and DP2 3rd plural, in German root clauses DP2 agreement was virtually categorical (92%); the lowest rate in root clauses was 62% (Dutch). DP2 agreement was also robustly attested in all four languages in embedded clauses, although at lower rates for all but Dutch, ranging from 88% for German to 46% for Faroese^14^.

The possibility of DP2 agreement is especially interesting from a theoretical point of view when it comes to person agreement: as outlined above in section 2, person agreement has been claimed to be universally restricted to be impossible downwards (see most prominently Baker's *Structural Condition on Person Agreement*, Baker, 2008, p. 52, discussed below in section 5.1). DP2 agreement in person in SCCs would therefore constitute an important counterexample to the universality of this claim.

In order to be able to show that agreement with DP2 is indeed downward agreement, however, we need to make sure that DP2 is indeed in a position that is below the agreement probe. This issue comes up in different environments in the languages we tested. As all four are V2 languages, the position of DP2 in root V2 clauses is not necessarily a position below the agreement probe: given den Besten's widely adopted analysis that V2 orders involve the verb in a high position in the left periphery (e.g., the Complementizer position) and the initial XP in the specifier of that position, one derivation for a clause like German (41) would have the initial nominal topicalising from a low predicate position, and the second nominal occupying a position above the agreement probe in T.


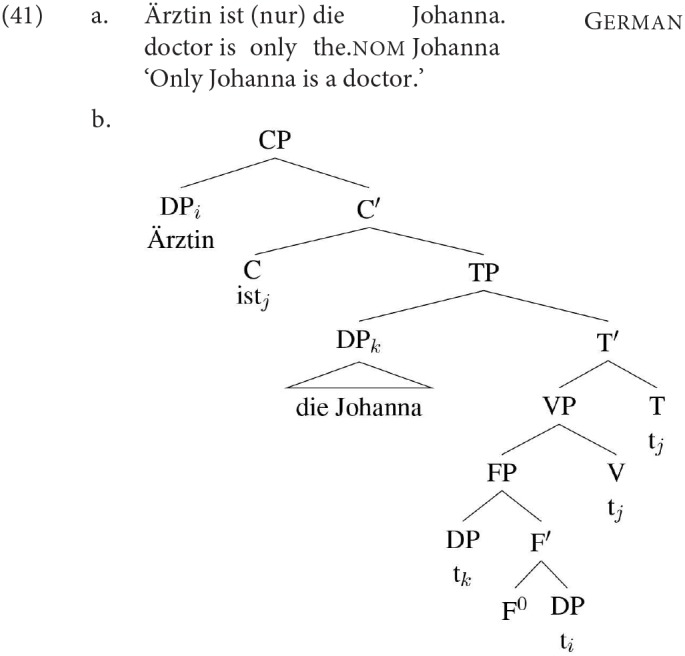


In the example in (41) it is clear that this is not a specificational sentence, rather the initial nominal is actually a predicate that has presumably reached the initial position in the clause by A′-movement, given that it is a bare (determinerless) nominal of a type that typically cannot appear in subject position (compare for example the ungrammatical case in (42), which is unacceptable because the bare NP Ä*rztin* cannot function as the subject of *reich* '*rich*', just like e.g., *mayor* or indeed *doctor* in English):


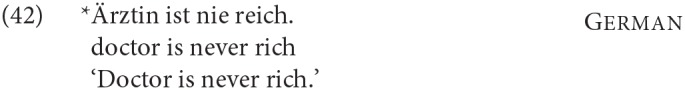


The issue is potentially more complicated with the specificational structures we are dealing with, however. Because, as discussed above, definites can also get a predicative interpretation (as in e.g., *Joan is the best-paid psychiatrist in Europe* and its translation equivalents), root sentences corresponding to English (39b) on the one hand, which involve predicate topicalization (A′ movement) and to (39a) on the other, which instantiate a specificational structure, are string-identical in the languages we looked at.

It is however possible to establish that the DP2 agreement that we find in specificational sentences in Faroese, Icelandic, Dutch and German is not simply reducible to the result of the kind of A′ predicate fronting + V2 illustrated in (41a), although the situation is most difficult in German. The type of A′ predicate fronting just discussed is generally taken to be a root phenomenon (Heycock and Kroch, 1998; Heycock, 2012), and in a V2 language is thus expected to pattern together with V2 order. In the SOV languages Dutch and German, the embedded clauses in our materials were all verb-final, and hence unambiguously had no possible parse as embedded V2. In the SVO languages Faroese and Icelandic, embedded V2 has been shown to be possible in environments that allow “embedded root phenomena,” but it is not freely available across all clause types. In order to avoid the confound of a parse as embedded V2, our materials therefore had the copular clause as an embedded interrogative (introduced by the equivalent of *whether*), as interrogatives are known to be the least favorable environment for embedded V2 (see e.g.,Thráinsson, 2007 p. 44 for Icelandic, Heycock et al., 2010 for Faroese and Icelandic). In some of our experiments/conditions for Faroese and Icelandic we added a further control, namely the inclusion of sentential negation. As discussed extensively in Mikkelsen (2002) for Danish, negation in Faroese and Icelandic occupies a position somewhere at or just above the left edge of the VP. Hence, if DP2 follows negation, it must be in a low position, not in the specifier of TP. We tested the influence of negation in the Faroese and Dutch production studies, with the result that negation did not affect agreement patterns (see Hartmann and Heycock, 2018e, 2019 for details), supporting the assumption that in these embedded clauses the first DP below the complementizer is indeed parsed as the subject, and DP2 as occupying a lower position^15^. We did not include negation in all of our experiments/conditions, since the examples were already fairly complex and adding negation to the interrogative adds further complexity.

While, as mentioned above, it is straightforward in the SOV languages Dutch and German to construct clauses that are unambiguously non-V2, these languages present a different issues in that they both, in some circumstances, allow “scrambling” to front a constituent all the way to the left of a subject in an embedded clause, and it has been argued that such scrambling is also an instance of A′ movement (see Neeleman and van de Koot, 2002, 2008; Grewendorf, 2005; Frey, 2010 for discussion and references). For these two languages, then, such a derivation might potentially be another confound. In Dutch this is not a serious concern. Such scrambling is much more restricted than in German: this kind of A′-scrambling requires a very specific context and is not even accepted by all speakers (see Neeleman and van de Koot, 2002, 2008). Additionally, in some of the conditions/experiments we added negation (which marks the edge of the VP) to further support the parse in which DP2 is in a low position. In German, scrambling of an object across a subject is more freely available than in Dutch, making a parse in which DP2 occupies the subject position (above T) with DP1 in a higher A′-position possible. In such a structure, DP2 agreement is not necessarily downwards agreement. However, A′-scrambling of a non-referential DP1 across DP2 in a copular clause is also an information-structurally restricted option even in German, i.e., it only occurs in contrastive/focus/emphasis contexts (see Frey, 2010 for discussion and references). As the sentences were presented out of the blue without such a context, it is unlikely that participants ended up analysing our test sentences as cases where a predicate has undergone A′-movement to a high position in the left periphery. Therefore, while acknowledging that we cannot completely exclude the possibility of an alternative parse, we think that DP2 agreement in German SCCs is also most plausibly taken to be an instance of downwards agreement.

In sum, we think that the materials we tested across the four languages indeed represent structures in which DP2 occurs in a position below the agreement probe. In this light let us now turn to the results.

In all four languages, in non-V2 contexts, DP2 agreement occurred (in production) and was judged relatively acceptable in the rating tasks, although as we have documented, the rates of production and the degree of acceptability was not the same across languages. We will discuss the differences in section 5 and concentrate on the shared production results here.

As mentioned at the beginning of this section, speakers of all four languages produced DP2 agreement when the two DPs were mismatched for number in a V2 clause like (43a). Crucially, this was also the case in embedded contexts, (43b). The rates of production for examples like (43) are given in Table 1. Note that in all the tables, where we give information about DP2, “3pl DP” means that the nominal in question is a “full” (non-pronominal) plural DP.

**Table 1:** Production of DP2 agreement in % (DP2 is a non-pronominal, full DP).

**Context**	**DP2**		**Dutch**	**German**	**Faroese**	**Icelandic**
Main Clause	3pl	DP	62%	92%	64%	74%
Embedded Clause	3pl	DP	70%	88%	46%	66%


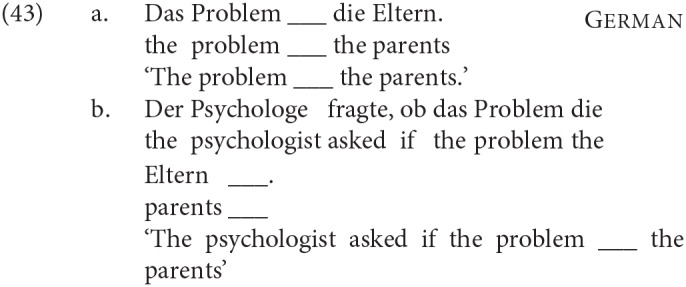


While there are differences between number and person (see section 5) all four languages also show the production of DP2 agreement when DP2 is non-third person as in (44), see Table 2^16^.

**Table 2:** Production of DP2 agreement in % (DP2 is a pronoun).

**Context**	**DP2**		**Dutch**	**German**	**Faroese**	**Icelandic**
Embedded Clause	2sg	Pronoun	97%	(99%)^*^	12%	48%
Embedded Clause	2pl	Pronoun	98%	(98%)^*^	66%	68%^**^

* *German data is based on main clauses*.

** *This includes cases of 3pl (number-only agreement) and 2pl (full agreement in both number and person) marking: see section 5.3 for this distinction*.


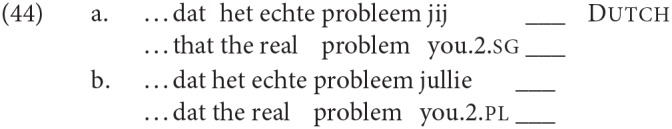


So from the production data, we conclude that DP2 agreement is a viable option for at least some speakers, in all four languages. The production data show, however, that there is significant variation with respect to the extent to which DP2 agreement is a possible or preferred option. This is also reflected in the rating data that we obtained for DP1 and DP2 agreement both for number agreement (with DP1 and DP2 differing in number only) and person agreement (where DP2 is a 1st or 2nd person pronoun). See Table 3^17^^,^^18^. In this table we see that there are also differences between languages in the rating advantage of DP2 agreement. So while the difference in German is 0.94 in embedded clauses with 2pl pronouns, in Faroese it only reaches 0.33 for the same case. In Icelandic on the other hand, overall, speakers rated DP1 agreement higher than full agreement (in both person and number) with DP2^19^. We nevertheless find a small group of speakers who rate DP2 agreement over DP1 agreement (see Hartmann and Heycock, 2018d), supporting the conclusion that DP2 agreement for number and person is possible for at least some varieties of Icelandic (for differences in the extent to which DP1/DP2 agreement is possible in all four languages see section 5).

**Table 3:** Rating advantage of DP2 agreement (z-scores).

**Context**	**DP2**		**Dutch**	**German**	**Faroese**	**Icelandic**
Embedded Clause	3pl	DP	0.80	1.11	(0.22)^*^	–
Embedded Clause	2pl	Pronoun	0.35	0.94	0.33	−0.48

* *Faroese materials used 3pl pronoun rather than full DP*.

Thus the rating data also illustrates our point that the languages under consideration differ in how much variation they exhibit^20^.

### 4.2. Lack of Default Agreement

As discussed above, in a specificational sentence DP1 is always 3rd person. Because in many cases mismatches in ϕ-features can only be tested with DP1 being singular and DP2 being plural or non-3rd person, one might be tempted to analyse what we have been calling DP1 agreement rather as “default” 3rd person singular, or lack of agreement with any DP. However, there are configurations where it is possible to tease these possibilities apart. We set up such cases and found that, wherever we were able to test, default is not in fact an option in SCCs. This seems clear in English: if DP1 agreement in (45a) were default agreement, we would expect a 3sg copula also with two plural DPs, but this is sharply ungrammatical (see already Heycock, 2009 for English and Faroese).


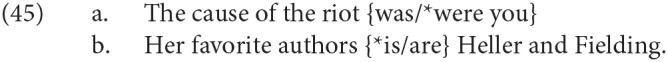


Initial informal evidence suggested already that default is not grammatical in SCCs in all the four languages in our studies. We included this configuration in our rating studies to get a value for a clearly ungrammatical agreement pattern with SCC (a baseline), see the examples in (46). The results showed indeed that default is ungrammatical: in Icelandic and Dutch default is significantly worse than any of the other conditions tested; in Faroese default is numerically but not significantly worse than DP1 agreement; in German, which is a consistent DP2 agreement language, default agreement is as bad as DP1 agreement, see Table 4, illustrated with respective examples in (46).

**Table 4:** Ratings in z-scores for default agreement in contrast to DP1 and DP2 agreement.

	**DP1**	**DP2**	**Verb**	**Dutch**	**German**	**Faroese**	**Icelandic**
Default	3pl	2pl	3sg	−0.78	−1.13	−0.79	−0.66
DP1	3sg	2pl	3sg	−0.67	−1.18	−0.73	0.34
DP2	3sg	2pl	2pl	−0.32	−0.24	−0.40	−0.14


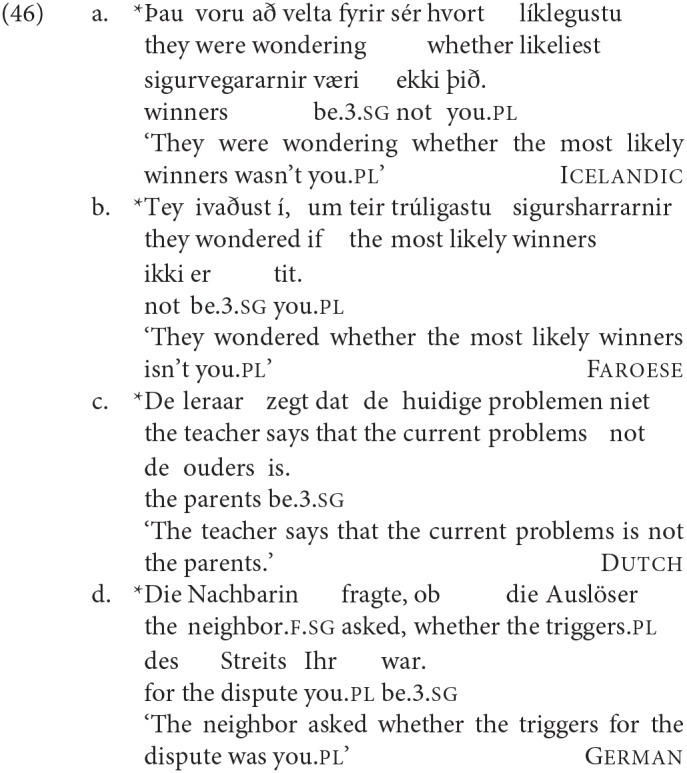


This further supports the conclusion from English that 3rd singular agreement with a 3rd singular DP1 is DP1 agreement, not default. Thus, any analysis in which DP1 does not have ϕ-features to be agreed with, see e.g., Béjar and Kahnemuyipour (2017), cannot be generally upheld.

### 4.3. DP1 Agreement in XP-Initial V2 Contexts

A further pattern that all four languages share is a specific effect in V2 contexts which we relate to C[omplementizer]-agreement. When considering adjunct-initial root clauses like (47) (which we will refer to as XP-initial V2 clauses in the tables below) in the four languages we discuss, we see that the production rate of DP2 agreement drops significantly in all four languages. This drop is especially striking for German, where we otherwise found a rather stable and strong preference in production for DP2 agreement across all other contexts we tested.


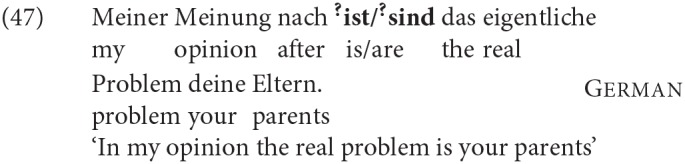


This difference also shows up in acceptability ratings, although the effect seems less dramatic, for reasons that we do not yet understand. As can be seen in Table 5, in the production data the adjunct-initial V2 order results in a “flip” from DP2 agreement to DP1 agreement being the most frequently produced order in all four languages (for all but German in fact the production of DP1 agreement in this order is close to categorical). Table 6 shows the extent to which DP2 agreement is rated higher than DP1 agreement in the judgment task in three different environments, including the adjunct-initial V2 order, In the rating data for Dutch, the “flip” in production corresponds to slightly—but significantly—higher ratings for DP1 agreement over DP2 agreement in the adjunct-initial order only. In the German rating data, the advantage of DP2 agreement in adjunct-initial root clauses is significantly reduced compared to the advantage of that agreement in embedded clauses and DP1-initial main clauses, so that in adjunct-initial V2 clauses there is no significant difference between the ratings for DP1 and DP2 agreement^21^.

**Table 5:** Production of DP2 agreement in % in different V2 (root) contexts.

**Context**	**DP2**		**Dutch**	**German**	**Faroese**	**Icelandic**
DP1-initial V2 Clauses	3pl	DP	62%	92%	64%	74%
XP-initial V2 Clauses	3pl	DP	8%	29%	4%	2%

**Table 6:** Rating advantage of DP2 agreement (z-scores) for Dutch and German.

**Context**	**DP2**		**Dutch**	**German**
Embedded clauses	3pl	DP	0.48	0.57
DP1-initial V2 Clauses (Root)	3pl	DP	0.29	0.90
XP-initial V2 Clauses (Root)	3pl	DP	−0.30	0.12

Evidently, in these V2 languages one effect of the XP-initial order is that the order of the finite verb (in the cases we tested, always the copula itself) and the DPs becomes V_*fin*_ < DP1 < DP2. An initial hypothesis might therefore be that the increased advantage for DP1 agreement here is some kind of performance effect tied to the linear order. However, whether or not there is a performance effect contributing to the increased production/acceptability of DP1 agreement, it does not seem likely that this is an effect which produces DP1 agreement *in a system in which DP1 agreement is ruled out by the syntax*. This is particularly relevant for German, where our results based on other configurations suggest that DP1 agreement is essentially ungrammatical. Consider, for example, that given the relatively free word order of German, it is possible for the DP immediately following the finite verb in an XP-initial non-copular sentence to be the object, even though the “default” order would be for this position to be occupied by the subject:


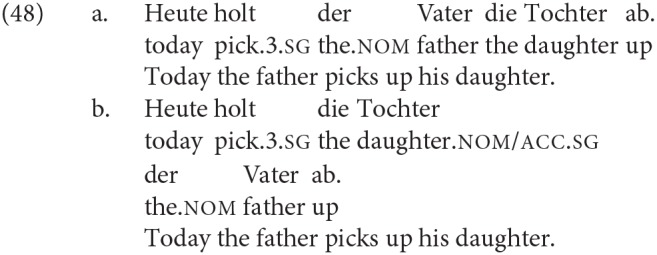


As there is syncretism between feminine singular nominative and accusative, in (48b) it is only when the unambiguously nominative singular DP *der Vater* is reached that it becomes evident that *die Tochter* cannot be the subject. One can reproduce such a structure with plural noun phrases, which are equally syncretic for nominative and accusative. If there is a performance effect that induces agreement with the first DP following the finite verb in second position, we would expect to find that examples like (49) are both produced and judged grammatical:


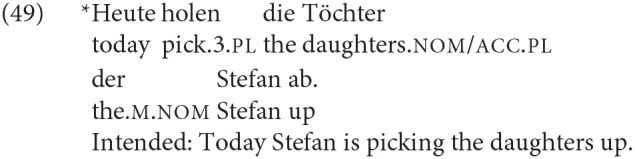


In the judgment of the German-speaking author of this paper, (48b) rather has the effect of a garden-path sentence, and (49) is simply unacceptable. This seems quite different to what is observed with the XP-initial copular clauses with DP1 agreement.

We have therefore pursued a different approach to explaining agreement in the adjunct-initial order. Namely, we have argued that what we observe here is not regular subject-verb agreement, associated with an agreement probe in T; instead we propose that here the agreement on the verb is in fact the exponent of a probe on C, which agrees with the closest DP in its c-command domain. As mentioned above in section 2, it has been known for some time that complementizers in Germanic sometimes also carry agreement features. The case mentioned above was so-called complementizer agreement, which is particularly associated with West Germanic varieties, see Bayer (1984), Ackema and Neeleman (2004), van Koppen (2005), and van Koppen (2017) among many others. An example from a Dutch variety was given above as (8), (50) is a further example, this time from Flemish, where the complementizer *dat* in (50) is inflected for number and person in agreement with the subject that immediately follows it.


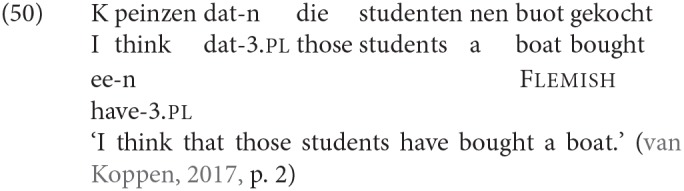


Note that this type of complementizer agreement only obtains when the subject immediately follows the complementizer in the linear order.

A second type of C-related agreement occurs in cases of so-called “inversion agreement” in Dutch. The distinct marking of the 2nd person singular in Standard Dutch is obligatorily omitted when the 2nd person subject immediately follows the finite verb in exactly the kind of adjunct-initial V2 structures where we find the unexpected high rates of DP1 agreement in our copular clauses (for a discussion of inversion agreement in other varieties of Dutch see Don et al., 2013 and references therein):


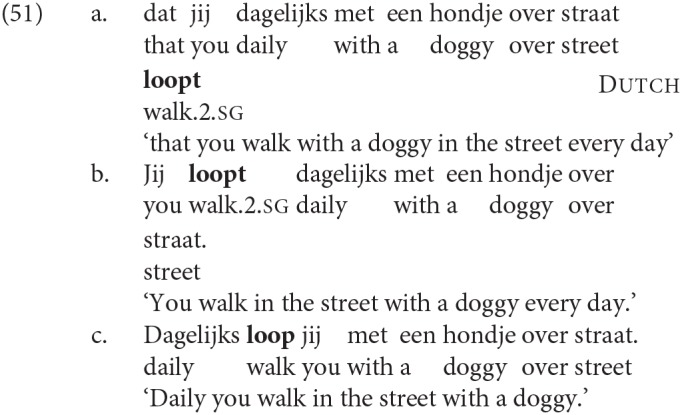


As just noted, both types of C-related agreement discussed in the literature only obtain when the DP immediately follows the C position (see the proposals in the literature referenced in van Koppen, 2017 on how this adjacency requirement can be implemented). This is exactly the configuration in which we found increased production/acceptability of DP1 agreement in the specificational sentences. Given this parallel behavior, we analyse the significant increase in the use/rating of DP1 agreement in SCCs when the copula has moved to C and is immediately followed by DP1 as the result of a type of inversion agreement. The agreement probe on C—to which the finite verb has moved—probes downwards and finds the closest available target, which is DP1, as in (52)^22^.


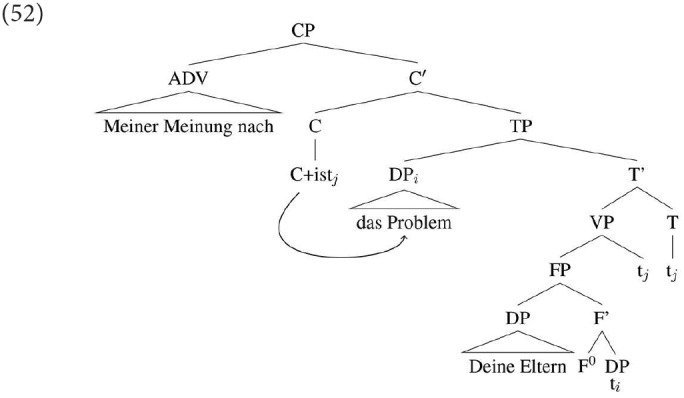


Thus, the agreement on the verb is the exposition of agreement of a person/number probe in the C-domain, whereas agreement in the T-domain is not expressed; here we have to assume that when both C-agreement and T-agreement conflict, but have to be realized on a single head (the verb), the conflict is resolved in favor of C agreement for most speakers^23^. In most of our languages this C-agreement is not usually visible, because in most sentences no differences between agreement in the T-domain and C-domain can arise with usually only a single nominative argument being present, see Hartmann and Heycock (2018b) for details.

## 5. Fine-Grained Differences: Number vs. Person

### 5.1. Background on Person vs. Number Agreement

Work on agreement especially in the last 15 years has drawn attention to the fact that agreement for person and agreement for number are not fully parallel. This topic is explored in depth in Baker (2008), Preminger (2011), Preminger (2014), and Ackema and Neeleman (2018) among many others (see in particular the references in Ackema and Neeleman, 2018). In general, person agreement is more restricted (in the terms of Baker, 2008 more “fragile”) than number agreement. In the Germanic family that we are concerned with here, the most prominent case comes from Icelandic. As mentioned earlier, Icelandic has a number of verbs whose subject has to appear with dative case-marking^24^. Some of these verbs are transitive, and have their lower argument appear in the nominative case; another class consists of verbs that select for non-finite clauses of one type or another, with the subject of the embedded non-finite clause again appearing in the nominative. Strikingly, the finite verb may agree in number with the low nominative argument (as seen in the (a) examples), but not in person (as seen in the (b) examples).


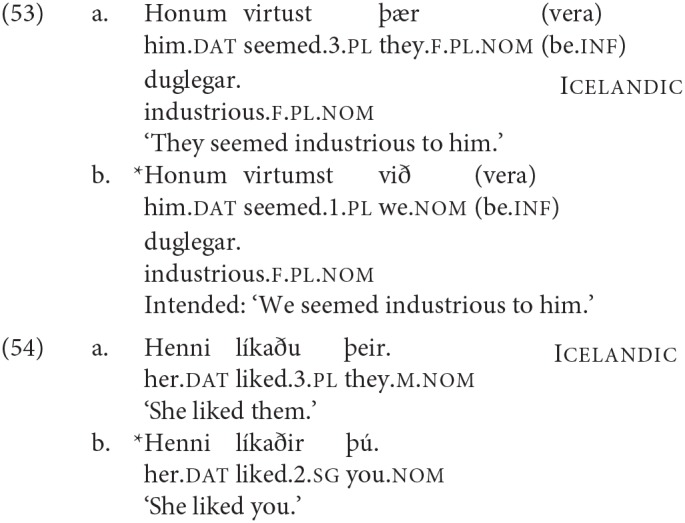


As noted earlier with respect to (6), an additional complexity here is that “default” 3rd singular agreement rescues examples like (53b), where the nominative argument is not an argument of the higher clause, but does not have the same effect—at least for many speakers—on examples like (54b):


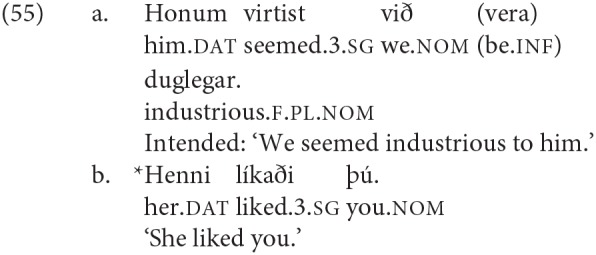


There are a range of suggestions as to how to account for such restrictions in general, and the Icelandic case in particular. One prominent approach is that of Baker (2008), where it is claimed that while number agreement can obtain “at a distance,” this is ruled out for person agreement, which can only be established via a specifier-head relation, as expressed more formally in the SCOPA:


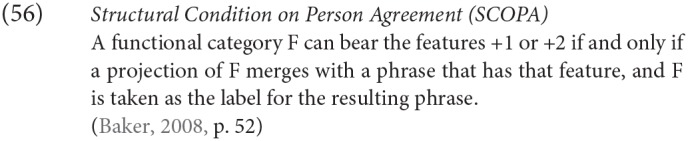


An alternative family of proposals relates the “person effect” seen in this configuration in Icelandic to a constraint observed in combinations of direct and indirect object clitics in a number of languages, the Person Case Constraint. This type of proposal is built on two core ideas. First, it is argued that a significant set of cases where there is a “low” 1st/2nd person argument that gives rise to ungrammaticality [including Icelandic examples like (54) and (53)], a higher argument intervenes between the agreement probe and the 1st/2nd person argument, preventing agreement from being established with that lower argument. In the case of the Icelandic dative-nominative constructions, this intervening argument is the dative DP. Second, 1st and 2nd person pronouns have the special property that they need to be licensed via agreement with a relevant probe, see Béjar (2003) and Béjar and Rezac (2003) for key proposals, and Preminger (2014) for a recent discussion of Icelandic cases like the ones just presented. This special property of 1st/2nd person pronouns is summed up in the Person Licensing Condition (PLC) of Béjar and Rezac (2003).


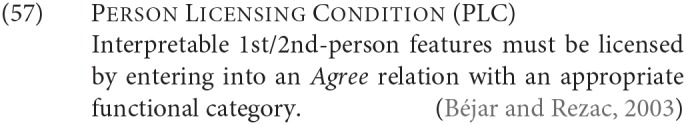


This formulation is subsequently amended in Preminger (2011) to exempt person features in clauses—even small clauses—without person ϕ-probes, precisely to account for the grammaticality of examples like (55a):


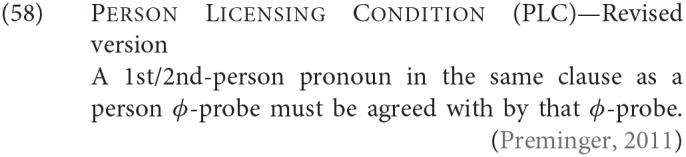


The net effect is that when some nominal intervenes between an agreement probe on some functional category and a 1st or 2nd person pronoun, the probe will fail to “reach” the pronoun (intervention) and the resultant lack of agreement will be fatal (PLC). More has to be said about why agreement for number with a “low” nominal (3rd person pronoun or nonpronominal DP) is possible even in the presence of an apparent intervenor; we leave this aside here, but see the cited works for details.

A third option for deriving the restrictions on person agreement also relies on the fact that such cases involve an “intervening” nominal, but assumes that agreement can be established with both DPs in such cases (“multiple agreement”). Ungrammaticality arises if there is no possible morphological exponent that is consistent with both of the agreement features that are copied onto the agreeing head. Thus it is argued that in (54b) and (53b) the dative argument triggers default (3rd person) agreement, and the nominative triggers 2nd or 1st person, respectively. The resulting conflict in feature values can however be resolved if there is a morphological form that happens to be syncretic for the two distinct values. Thus for example (59) is argued to be grammatical in Sigurðsson and Holmberg (2008) because for the verb *virðast* ‘seem' there is syncretism in the plural between 2nd and 3rd person:


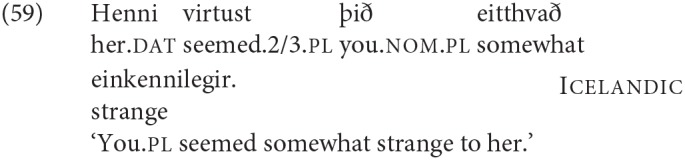


See Schütze (2003) (based on data from Sigurðsson, 1996) and Sigurðsson and Holmberg (2008) and Ackema and Neeleman (2018) for this kind of proposal for Icelandic Dative-Nominative structures^25^.

All the proposals just listed have been argued to be general restrictions on person agreement. It then becomes relevant to ask whether their effects are evident also in SCCs—and not only in Icelandic. That is, we might expect that agreement with DP2 should be possible only for number, and not person; and that failure to agree with a 1st or 2nd person DP2 should result in ungrammaticality. And indeed such a claim is made for Dutch in den Dikken (2019), on the basis of his own judgments. However, our data suggest that other Dutch speakers show a different effect, as will be made clear in the next sections.

### 5.2. Person Agreement in SCCs

First, as reported above in Table 2, DP2 agreement in person is produced in all four languages, though to varying degrees.

German is in general quite consistent in having agreement with DP2, as shown in Table 7^26^. In addition, the rating data show a consistent overall higher rating for DP2 agreement in this language^27^.

**Table 7:** Agreement patterns in SCCs in German.

			**Production**			**Rating (z-scores)**		
**Context**	**DP2**		**DP1**	**DP2**	**%DP2**	**DP1**	**DP2**	**DP2 advantage**
Main clause	3pl	DP	10	129	92%	−0.48	0.42	0.90
Embedded clause	3pl	DP	16	117	88%	−0.54	0.03	0.57
	2sg	Pronoun	1	158	99%	–	0.03	–
	2pl	Pronoun	3	131	98%	−1.18	−0.24	0.94
	3pl	DP	–	–	–	−0.73	0.48	1.11
			(Main clauses)			(Embedded clauses)		

Dutch is clearly different from German in a number of respects: see Table 8. Observe that more Dutch speakers than German produced DP1 agreement in number (where DP2 was a plural non-pronominal DP, Dutch speakers produced DP1 agreement in 38% of root clauses, and 30% of embedded clauses; the corresponding figures for German are 8% and 12%). However, in both languages we tested embedded clauses with 2nd person pronouns (both singular and plural) as DP2: in these cases the rate of DP2 agreement in Dutch rises to match that of German. The rating studies reveal though that in Dutch ratings drop in general when DP2 is a personal pronoun, independent of whether there is syncretism with 3rd person in the verbal agreement, or indeed whether the pronoun is 2nd or 3rd person. Thus there is no difference in the ratings between the conditions in (60a) and (60b) and the very small difference to (60c) is not significant. On the other hand, there is a significant difference between the cases where DP2 is a pronoun (whether 2sg, 2pl, or 3sg) and those where it is a full DP, as in (60d). In German we also see a difference between the ratings for cases where DP2 is a full DP, and those where it is a pronoun (2sg or 2pl), with the full DP condition rated more acceptable overall. However, we do not have a direct comparison within a single experiment that compares a full DP with a third person pronoun as DP2. Thus, our data cannot be used to argue for a pronoun effect in German. Informal discussions with native speakers of German and Dutch seem to suggest that there is indeed a difference between Dutch and German in that focused pronouns in SCCs are problematic in Dutch, but not in German: this clearly requires further investigation.

**Table 8:** Agreement patterns in SCCs in Dutch.

			**Production**			**Rating (z-scores)**		
**Context**	**DP2**		**DP1**	**DP2**	**%DP2**	**DP1**	**DP2**	**DP2 advantage**
Main clause	3pl	DP	69	113	62%	−0.54	−0.25	0.29
Embedded clause	3pl	DP	53	127	70%	−0.76	−0.28	0.48
Embedded clause	2sg	Pronoun	6	211	97%	−0.71	−0.30	0.41
Embedded clause	2pl	Pronoun	2	146	98%	−0.67	−0.32	0.35
Embedded clause	3pl	DP	–	–	–	−0.52	0.22	0.80
Embedded clause	3sg	Pronoun	–	–	–	−0.38^*^		–

* *The final row shows the rating where DP2 is a 3rd singular pronoun, and agreement is 3rd singular*.


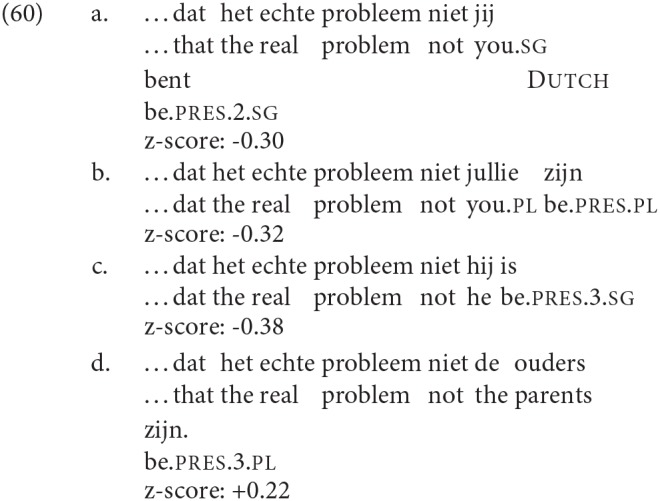


Thus, in our data, Dutch DP2 agreement in SCCs exhibits a pronoun effect and there is no evidence for a person effect.

From the perspective of whether or not downwards person agreement with a low nominative is possible in these languages (contra SCOPA), we need to look at the data quite carefully, and take into consideration independently known facts about these languages. German shows DP2 agreement both with number and person. On the face of it, this looks like a clear case of downwards person agreement into the VP. However, of the languages we are considering, German has the most “free” word order within TP; most relevantly here, object pronouns generally move out of the VP to the left edge of the “middle field”, which could be—depending on the analysis of scrambling—outside the c-command domain of the agreement probe (presumably T, but see footnote 22), as in (62):


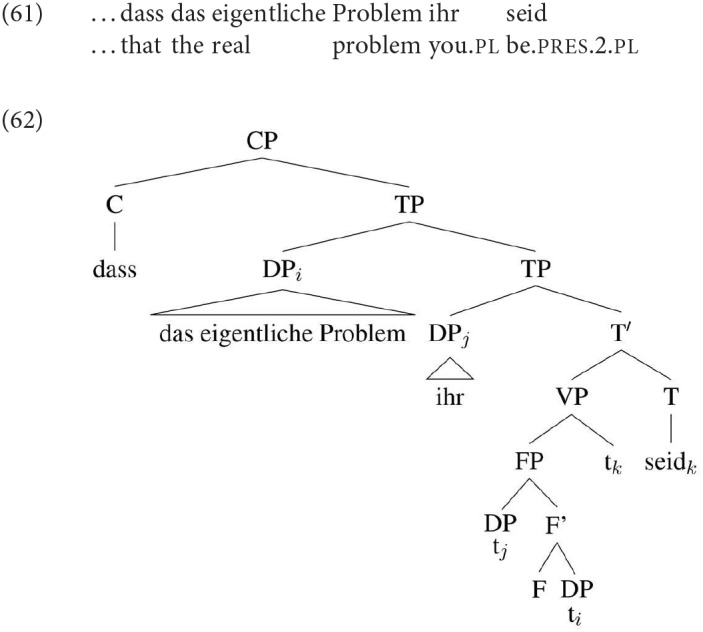


If speakers parse our copular sentences with pronominal DP2 as involving such movement, these examples would not necessarily involve agreement into the VP, and could not be used as an argument against theories that treat downwards agreement for person as impossible (as e.g., Baker's SCOPA) However, the alternative parse with the nominative DP2 in a low position is at least equally plausible: leftward movement is not obligatory, and focused pronouns in particular tend to remain within the VP. In an SCC the second DP is obligatorily focussed, so it is at the least possible that the pronominal DP2 has indeed remained in a low position. The usual way to force such a parse is to include negation, which would precede an unmoved, “low” pronominal. While we did not include negation in our materials for German, informally elicited judgments from native speakers informants suggest that the presence of negation does not affect the preference for DP2 agreement in any way.

In Dutch, the production data in Table 8 show that when presented with SCCs with a 2nd person pronoun as DP2, participants overwhelmingly chose to agree with the pronoun, regardless of whether this agreement was syncretic with 3rd person (97%–98%). Evidently, this is consistent with downwards person agreement being grammatical in this language. It is the case that, as we just saw, in Dutch, pronouns in general are less acceptable in the low position following negation and it could be objected that this production task does not allow us to determine whether speakers simply found this order unacceptable, and that they were making agreement choices for sentences that were ungrammatical for them. If we inspect the ratings data in the same table, however, we can see that while the ratings for the examples with pronouns were low, they were not at floor. Further, we find no additional effect of 1st/2nd person. That is, if there was a “person effect” on top of the pronoun effect, the ratings should be worse for 1/2 person pronouns than for 3rd person pronouns, but that is not what we see. Thus, we find no “person effect” in Dutch either, and we have evidence that person agreement with low nominatives is possible in this language.

We can strengthen this point by looking at the data in Faroese and Icelandic, in Tables 9, 10, respectively. Both languages are VO, so DP2 is clearly in a low position in the embedded interrogatives that we tested, since it follows the verb. In production in Faroese, we see that native speakers produce DP2 agreement to a significant extent (see section 5.3 below, and also Hartmann and Heycock, 2018e, for discussion of why DP2 agreement appears to be produced at an unusually low rate just when DP2 is the 2nd singular pronoun). In the rating data, we see that they in general prefer DP2 agreement over DP1 agreement, though ratings in general are rather low for SCCs with pronominal DP2. In Faroese there is some evidence that the rather low ratings when DP2 is a pronominal is not due to a person effect, since the ratings when DP2 is a 3rd person pronoun are not significantly higher than the ratings when it is 1st or 2nd person. It could be that there is a “pronoun effect”, as in Dutch, but to establish this would take further research, as the Faroese rating experiment did not include a condition with a non-pronominal DP2.

**Table 9:** Agreement patterns in SCCs in Faroese.

			**Production**			**Rating (z-scores)**		
**Context**	**DP2**		**DP1**	**DP2**	**%DP2**	**DP1**	**DP2**	**DP2 advantage**
Main clause	3pl	DP	18	32	64%	–	–	–
Embedded clause	3pl	DP	20	17	46%	–	–	–
Embedded clause	2sg	Pronoun	100	14	12%	–	–	–
Embedded clause	1sg	Pronoun	–	–	–	−0.56	−0.40	0.16
Embedded clause	2pl	Pronoun	39	76	66%	−0.73	−0.40	0.33
Embedded clause	3pl	Pronoun	51	54	51%	−0.66	−0.44	0.22

**Table 10:** Agreement patterns in SCCs in Icelandic.

			**Production**			**Rating (z-scores)**		
**Context**	**DP2**		**DP1**	**DP2**	**%DP2**	**DP1**	**DP2**	**DP2 advantage**
Main clause	3pl	DP	50	139	74%	–	–	–
Embedded clause	3pl	DP	63	123	66%	–	–	–
Embedded clause	2sg	Pronoun	109	99	48%	–	–	–
Embedded clause	2pl	Pronoun	see Table 11			0.34	−0.14	−0.48
Embedded clause	3pl	Pronoun	74	143	66%	–	–	–
Embedded clause	3sg	Pronoun	–	–	–	0.43		

In Icelandic, we see that overall, DP2 agreement is preferred with number agreement, but not person agreement, which is also reflected in the rating data, where DP1 agreement is preferred over DP2 agreement. Despite this fact, a more detailed investigation into the data reveals is that there are still some speakers in Icelandic (though few in our sample) who consistently prefer DP2 person agreement over DP1 agreement: for this see Hartmann and Heycock (2018d).

In summary, we do not find any clear evidence in SCCs of the kind of “person effect” (ungrammaticality of “low” 1st/2nd person nominatives) that is present in Icelandic dative-nominative constructions. To the extent that these languages allow for pronouns to appear as DP2, agreement is possible regardless of person.

It is also relevant to consider the production and rating of DP1 agreement. In Icelandic, the production and grammaticality (at least for some speakers) of DP1 agreement when DP2 is a 1st or 2nd person pronoun constitutes an argument against the general applicability of the requirement for 1st/2nd person pronouns to be agreed with (the Person Licensing Condition (PLC) described in section 5.1 above). It is clear from the Icelandic data that DP1 agreement is a viable option and the preferred option for many speakers in our sample.

Finally in our data we did not find any evidence for a syncretism effect that could be taken as evidence for multiple agreement in SCCs (recall the discussion in section 5.1 of multiple agreement as an account of the person effect in dative-nominative constructions in Icelandic). In all languages we tested syncretic forms (German: 1/3 plural, Dutch 1/2/3 plural, Icelandic: 1/3 singular, Faroese: 1/2/3plural) as potentially providing evidence for multiple agree, but either we found that syncretism did not have a significant effect (German, Dutch, Icelandic) or that what looks like a syncretism effect in Faroese, akin to what is found in dative-nominative constructions in Icelandic, in fact has a ratings profile that requires a different explanation (see Hartmann and Heycock, 2018e, and below). Thus, multiple agreement does not arise where there are two nominative arguments in a single clause (though it is a viable analysis for the dative-nominative construction, as we argue in Hartmann and Heycock, 2018d)^28^.

### 5.3. Person and Number Are Separate Probes in Icelandic and Faroese

There is one further aspect in the agreement domain in which we find variation in the languages under consideration that we wish to present here: Icelandic and Faroese show evidence that person and number are actually distinct heads. In Icelandic—where the distinction between the two probes has been made previously based on the pattern of agreement in dative-nominative cases, see Sigurðsson and Holmberg (2008)—we find direct evidence for this. In the production test in Icelandic we presented speakers with sentences where DP2 could differ from DP1 in number, in person, or in both, as illustrated in (63)^29^:


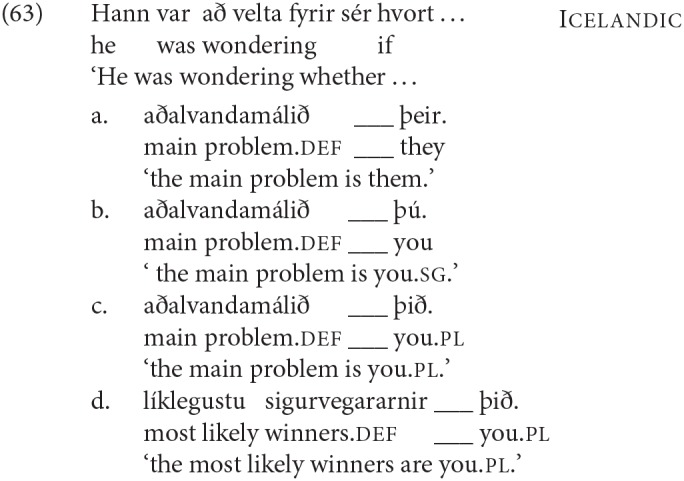


The choices made by the participants are tabulated in Table 11.

**Table 11:** DP1 vs. DP2 Agreement per condition in Icelandic (irrelevant cases excluded).

	**DP** **ϕ-features**		**Copula agrees with**			
**Cond**	**DP1**	**DP2**	**DP1**	**DP2 (all)**	**DP2 (Nr only)**	**Total**
A	3sg	3pl	74 (34%)	143 (66%)	n.a.	217
B	3sg	2sg	109 (52%)	99 (48%)	n.a.	208
C	3sg	2pl	68 (32%)	80 (38%)	63 (30%)	211
D	3pl	2pl	118 (56%)	91 (44%)	n.a.	209

The interesting case is condition C. In German, the other of the four languages where 2nd person is distinctively marked in the plural, nearly all responses (98%) had 2nd person plural agreement in the corresponding condition (full DP2 agreement), with just a few choices of DP1 agreement, 3rd singular (2%)—see again Table 7. In this condition in Icelandic, however, just under a third of the responses consisted of the 3rd plural form instead of either 3rd singular (DP1 agreement)^30^ or 2nd plural (full DP2 agreement). Thus in these Icelandic responses we see agreement with DP2 in number (plural), but not person.

It was argued in Sigurðsson and Holmberg (2008) that Number and Person are distinct heads in Icelandic, with Person higher than Number (consistent with the morphology in Icelandic, where Person morphology on the verb is consistently outside Number morphology). Sigurðsson and Holmberg (2008) argue, on the basis of the variation of patterns in agreement in the kind of dative-nominative constructions discussed earlier, that with these additional heads come additional landing sites for movement. The spine that they propose for the clause in Icelandic can be schematized as follows, where we have numbered the potential landing sites for ease of reference^31^:


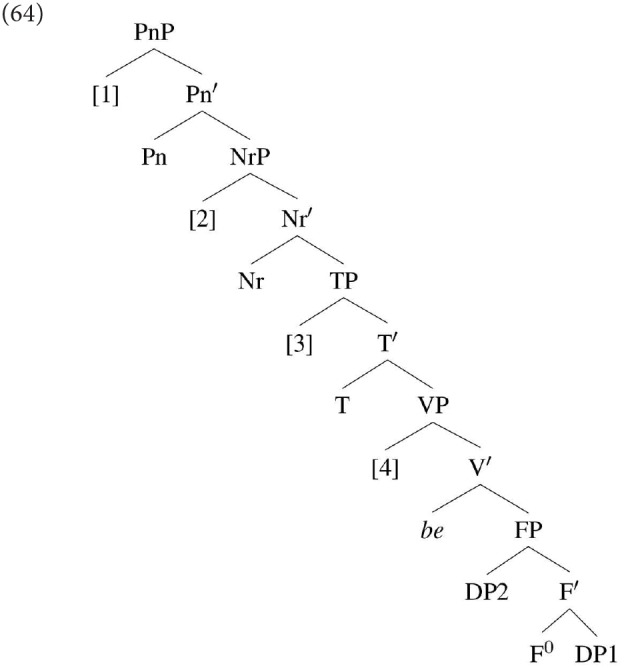


Importantly, there is a potential landing site for DP1 below Person but above Number [Position [2] in (64)]. If DP1 moves directly to position [1] above all agreement probes, the first DP that will be encountered by the agreement probes for both number and person will be DP2. This should result in full DP2 agreement. If DP1 moves rather to position [2] it will be accessible to the person probe, but DP2 will be found by the number probe. This should result in person agreement with DP1 (which in SCCs is always 3rd person) but number agreement with DP2. Thus this derives the Number-only DP2 agreement just described. Positions [3] and [4] as landing sites for DP1 will result in DP1 agreement in both person and number as in this case, DP1 is the closest target for both agreement probes. At present we do not see how this pattern of Number-only DP2 agreement could be derived by appealing only to the possibility of differential ϕ-sensitivity of the probe (Béjar and Kahnemuyipour, 2017).

It does have to be recognized that despite the robust production of this type of agreement in our experiment, in the ratings task it was rated rather low (z-score: -0.40), though it was significantly above the rating for default agreement (which was -0.66, see Table 4). There are two options why this might be the case. First, we only have a small number of speakers (4 to be precise) who rate the condition consistently (i.e., in all 3 occasions they rate it) above their average. Alternatively, we think that it is possible here that speakers are more aware of prescriptive pressures in the ratings task than in the production task^32^. Recall that in the case of number-only agreement, the morphology of the verb is neither a full match for DP1 or DP2. Clearly this is a rare configuration in the language, and although as far as we know there are no articulated prescriptions about agreement in specificational sentences in Icelandic, any speaker who is hesitant about the “correctness” of their response is unlikely to conclude that number-only agreement is the prescribed form. In the production experiment, speakers were never presented with forms of the copula: they generated these forms to fill the blanks. On the other hand, in the ratings task speakers were presented with examples in other conditions where the copula can be interpreted as agreeing fully with DP1 and/or DP2. We therefore suggest tentatively that speakers may be more conscious in the ratings task of alternative forms that seem more “standard” and that this may account at least in part for the rating of number-only agreement being lower than would be expected from its frequency in production.

In Hartmann and Heycock (2018e), we argue that Faroese also shows evidence that the person and number probes are on distinct heads in this language though the evidence is much less direct than in Icelandic, as number-only agreement in Faroese can conflate either with DP1 or full DP2 agreement. In the paper we show how the frequency differences and rating results are best understood in terms of two probes; below we will concentrate on the production data; readers are referred to the paper for full details.

In Faroese we tested agreement in the following three conditions^33^:


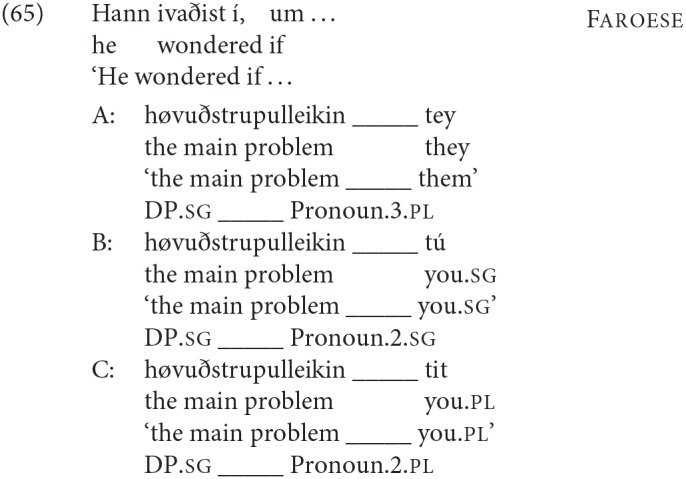


The results are tabulated in Table 12. It is important to bear in mind that Faroese has no distinct person marking in the plural on the copula, but 2nd person (and 1st in the present tense only) is marked distinctively in the singular, as illustrated in (66).


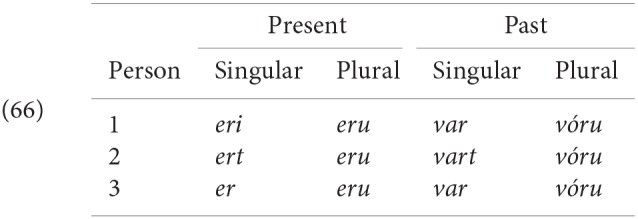


The observation of interest here is that the amount of DP2 agreement for person drops to 12% with 2nd person singular DP2, while it is much higher in the other two conditions^34^.

**Table 12:** Conditions and results of the Faroese production study on Person.

	**DP** **ϕ-features**		**Copula agrees with**			
**Condition**	**DP1**	**DP2**	**DP1**	**DP2**	**Total**	**%DP2 agreement**
A	3sg	3pl	51	54	105	51%
B	3sg	2sg	100	14	114	12%
C	3sg	2pl	39	76	115	66%

Once we take into consideration that person and number are separate heads, and as a result that number-only agreement is a viable option in Faroese, we can see why there is such a difference between production of apparent DP2 agreement in B on the one hand, and A and C on the other. As set out in Table 13, in both conditions A and C, number-only agreement [the agreement pattern associated with DP1 occupying position [2] in the tree in (64)] conflates with DP2 agreement (the agreement pattern associated with position [1]); in condition B, on the other hand, the agreement morphology associated with these two positions is distinct. Thus the apparent lower production of “DP2” agreement in condition B can be explained because it is the realization of only one possible configuration (DP1 occupying position [1]) while in conditions A and C, apparent DP2 agreement can be the realization of two configurations (DP1 occupying either position [1] *or* position [2])^35^.

**Table 13:** Agreement features, verb form and coding per DP1 position for Faroese.

			**Feature realization per DP1 position**			
**Condition**	**DP2 features**		**[1]**	**[2]**	**[3]**	**[4]**
A	3pl	V-features	3.pl	3.pl	3.sg	3.sg
		verb form	eru/vóru	eru/vóru	er/var	er/var
		coding	DP2	DP2	DP1	DP1
B	2sg	V-features	2.sg	3.sg	3.sg	3.sg
		verb form	ert/vart	er/var	er/var	er/var
		coding	DP2	DP1	DP1	DP1
C	2pl	V-features	2.pl	3.pl	3.sg	3.sg
		verb form	eru/vóru	eru/vóru	er/var	er/var
		coding	DP2	DP2	DP1	DP1

Thus, we conclude that both Faroese and Icelandic have person and number as separate heads, which provides an additional landing site for DP1. As a result, number-only DP2 agreement is a possible option, even though it might not be overtly marked in all cases.

### 5.4. Summary

Summarizing our findings and relating them to other works on agreement patterns in specificational copular clauses, we found that these patterns are due to general properties of the agreement system of each language and properties of SCCs. The relevant factors that we isolated are: (i) case (ii) structural configuration (iii) number of agreement probes; (iv) type of agreement probe.

- Reviewing previous literature, we pointed to one first relevant aspect for agreement, namely the case of the two DPs, and as a result their availability for agreement. In English, DP2 appears in accusative case, which makes it inaccessible as a controller of agreement in English. In the languages that we discussed, this is not an issue. In all four languages we looked at, both DP1 and DP2 are nominative and as such potential controllers of agreement.
- We have argued that the crucial source of variation in the Germanic languages arises from the SCCs being inversion structures, which creates a configuration in which the initially lower DP1 can become accessible to a higher agreement probe, because it moves above DP2 to become the highest DP below a yet higher probe or probes, see (64) above. This sets SCCs apart from predicational copular clauses, which do not show significant variation in agreement patterns in Germanic.
- A third relevant factor is the number and structure of agreement probes. This is relevant in the discussed languages for two effects we saw in the data. First, the separation of the number and person probe in the T-domain in Icelandic leads to a third possible pattern of agreement: number-only DP2 agreement. Second, it provides an explanation for an apparent increase in DP2 agreement in Faroese where this is indistinguishable from number-only DP2 agreement due to morphological syncretism.
- Additionally, we take the increase in the production and acceptability of DP1 agreement in all four languages^36^ to be due to an agreement probe in the C-domain. The effects of this probe are usually not visible, as there is typically just one target for agreement for both probes; they are manifest in specificational copular clauses because there are two.

## 6. Newly Opened Questions

Overall, we intend our work to contribute a new range of data relevant both to specific questions concerning copular clauses, and to more general questions about how agreement goals are “chosen” when there is more than one, and how apparent restrictions on person agreement might be explained. We have tried to highlight throughout how these new data bear on existing theoretical questions about agreement. At this point we would like to add the perspective of what new questions are opened up by this data that are relevant for future research in this domain.

First of all, we have shown for Dutch, Faroese, and Icelandic, that there are a range of agreement options that native speakers choose from. Considering the patterns that we have presented above, an important question that immediately arises is what independent factors determine the agreement options available in the different languages. Clearly the hypotheses that can be entertained depend on prior decisions regarding the most promising analyses of these different options. We have outlined two alternative types of analysis above. In one, initially set out in Béjar and Kahnemuyipour (2017, 2018), DP1 agreement arises in specificational sentences because DP1 in such sentences is ϕ-deficient and the agreement probe on T may be sensitive to exactly the feature or features that DP1 lacks, so that DP1 may be “skipped.” In the other, initially set out in Hartmann and Heycock (2016, 2017, 2018d,e, 2019), we have proposed that DP1 may “evade” agreement by moving directly to a position above the agreement probe (or, where Person and Number are not only distinct probes but distinct heads, above Number but below Person).

We take as a baseline assumption that the ϕ-features on DP1 in a specificational sentence do not vary between languages (*a fortiori* they do not vary between idiolects within a single language). Under the first type of analysis, then, the kind of variation between DP1 and DP2 agreement that we have documented here would have to reflect variation in the ϕ-sensitivity of the probe—intra-speaker variation, for many speakers of Dutch, Faroese, and Icelandic^37^.

Under the alternative approach that we have outlined, the observed variation has to be due to the possibility of DP1 making use of different landing sites. In Heycock (2012) it was proposed that direct movement above the agreement probe (taken in that paper to reside in T) may occur if the copula, rather than uniformly instantiating *v*, may instead instantiate T, which is then the lowest functional head above the small clause. This is evidently only a possible analysis for the finite copula, given that in all the languages under consideration here, and also in English, the copula may appear in non-finite contexts, for example below a modal. It was noted in that paper that initial results from Faroese indicated that exactly in such contexts, the production of DP2 agreement dropped sharply, to the point where a possible conclusion was that it was in fact ungrammatical. In our subsequent studies reported on here, we have not been able to include conditions testing for this kind of locality effect across the languages at issue: evidently this is a question that demands further research, if we are to be able to answer questions about locality effects in these structures and what they (or their absence) can tell us about the right analysis. As noted already in Heycock (2012), however, at least in German our impression, based on the German-speaking author's judgment and informally gathered judgment from other German-speakers, is that agreement with DP2 is possible even when the copula is embedded below e.g., a modal:


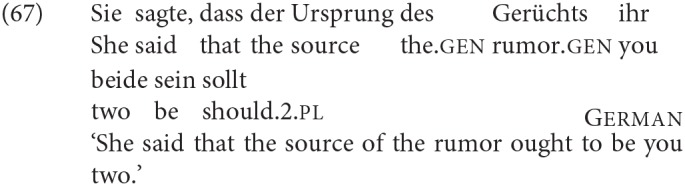


Taken together with the virtually categorical DP2 agreement (setting aside what we have analyzed as C-agreement) in German, this suggests that in this language there is never a landing site for DP1 below the agreement probe. This would be in line with the discussion in the literature showing that German provides hardly any evidence for a T projection independent of verbal projections including projections for auxiliaries and modals (see Haider, 1997; Sternefeld, 2009). If this is indeed the case, in German the agreement probe might in fact be on *v*-related heads. If these did not provide specifier positions—for a reason that would have to be determined—any landing site for DP1 would be above it.

The difference between German on the one hand and Dutch, Faroese and Icelandic then could be that the latter have a separate T projection. This has the effect that the edge of vP is a possible landing site for DP1, below the agreement probe. For many speakers of Faroese, Dutch, and Icelandic, of course, although DP1 agreement is possible, it is not the only option, and in this they differ from English. That is, the movement to the edge of vP is a possible option, but direct movement to the position higher than the agreement probe, directly to the subject position is also possible.

A second, closely related question, is what independent evidence a learner has for these differences between the languages. One potential factor in this is the role of the morphological exponence of agreement features. For example, we presented above evidence that Icelandic has distinct heads for the number and person probes. An obvious hypothesis is that this might correlate with a transparent morphological distinction between number and person morphology on the verb (as pointed out in Sigurðsson and Holmberg, 2008, Icelandic verbal agreement morphology systematically has distinct number morphology close to the verb stem, and then person morphology). However, we found evidence for a “split” probe also in Faroese, where there is no similar clear split between person and number morphology. Further, German has a morphological paradigm that is comparable to the one in Icelandic, but there is no evidence for split probes in this system. This suggests that any relation between the nature of the morphological expression of agreement and the range of options will not be a simple one, but more work is needed here.

A third question that arises in the context of our work concerns the question of what determines native speakers' choice of form when they have more than one option available. That is, what factors influence/determine the choice of variant when there is (at least the possibility for) intra-speaker variation? These choices might be influenced by factors such as formal/informal context, processing constraints, and the like. Speakers might also develop preferences based on features that are known to affect agreement in other languages: pronoun vs. full noun phrase, definiteness, information structure, animacy or even the task/goal for the expression used (see below) etc. In general, it seems to us that specificational copular clauses in Faroese, Icelandic, and Dutch provide an interesting new testbed for the study of syntactic/morphological variation/optionality.

A fourth question that arises in all cases of agreement is the role of linear order and the difference between “true” grammatical agreement and processing effects associated with linear order, for example the much-discussed case of “agreement attraction” (see among many other, Bock and Miller, 1991; Franck et al., 2002; Wagers et al., 2009; Patson and Husband, 2016 and references therein). For example, we found an effect in our production experiments concerning number that could potentially be a processing effect of distance: for all our languages, DP2 agreement decreases in V2 clauses when an adverbial intervenes between DP1 and DP2 compared to the same structures without an adverb, see Table 14.

**Table 14:** Production of DP2 agreement with and without intervening ADV % in V2 contexts.

	**Dutch**	**German**	**Faroese**	**Icelandic**
DP1 V DP2	62%	92%	64%	74%
DP1 V ADV DP2	37%	82%	42%	45%

This might in principle be an interaction of optionality and processing preference: when two options are grammatical, speakers might tend to choose the option which allows agreement with a more local DP. This is a possible explanation for Dutch, Faroese and Icelandic (where we have argued both DP1 and DP2 agreement are grammatical options), however, it leaves the effect in German unexplained (where only DP2 agreement is possible in the T-domain). Even though the increased use of DP1 agreement in German is smaller compared to the other languages, it is still significant, see the details in Hartmann and Heycock (2018b). It remains to be established whether this effect should be considered an effect of C-agreement or a processing effect with DP2 being linearly more distant. Establishing this would require cross-disciplinary work to tease apart the two types of effects.

Finally, our research also raises the question of the kind of evidence obtained from production and rating studies. As discussed in section 3.3, we used both methods in order to combine the merits of both (and control for the limitations of both). We did, however, find some mismatches between the data from the two types of study that we do not yet understand. For example, considering the production study in Icelandic, with a 2nd person plural DP2 (the third row of data in Table 11), the production of the three types of agreement (DP1, DP2, and person-only DP2) is roughly equal. In the rating data, however, DP1 agreement is rated significantly higher than either type of DP2 agreement. Part of this seems to be due to the fact that some speakers seem to prefer one or the other agreement pattern generally, and in the rating data, we had more speakers who prefer DP1 agreement over DP2 agreement. But further investigation into the effect of the task on the results seems necessary. The production task, which is effectively a forced-choice task as far as agreement is concerned, may be less sensitive to small differences in the materials, as the possible options for the participants are very limited. In the rating task, especially with such fine-grained methods such as the magnitude estimation and thermometer ratings, ratings might be more directly affected by small differences in the materials. Systematic consideration of these methodological effects therefore seems in need of further study.

## 7. Conclusion

In this paper we have provided an overview of agreement patterns in Specificational Copular Clauses in Germanic. Based on experimental work summarized here and reported in more detail in Heycock (2009, 2012), Hartmann and Heycock (2016, 2017, 2018b,d,e, 2019), we have attempted to synthesize the main insights and generalizations, and we have proposed an analysis of specificational clauses along the lines given in (64), but also discussed alternatives.

According to the analysis we have outlined, depending on the landing site of inversion of DP1 in a position below, above or between agreement probes (where they are split), different agreement arises. For the four languages under discussion we see the following patterns. First, all four languages under investigation show DP2 agreement to a greater or lesser degree, i.e., all four languages have [1] as a landing site for DP2. German is the one language that shows exclusively such agreement, potentially indicating the lack of the T-domain. Second, we find variation with respect to the other positions: Icelandic and Faroese have number and person split, so both languages have position [2] as one available option for speakers. This results in number-only DP2 agreement. Additionally, Icelandic, Faroese and Dutch allow for DP1 agreement (again to varying degrees), i.e., DP1 can land in position [3]/[4] below these agreement probes. Finally, we have isolated a further pattern of agreement which is located in the C-domain, so independent of the positions in the tree in (67). This C-related agreement appears in XP-initial V2 clauses in all four languages.

Overall we consider that agreement patterns in SCCs in Germanic help to understand SCCs as inversion structures, and provide further insight in factors that play a role for agreement within and across languages, namely the number of agreement probes and their location (in the C- or T-domain), the syntactic configuration, and the option of downwards agreement with a low nominative.

## Data Availability Statement

The overview provided in this paper is based on a series of experiments reported in Hartmann and Heycock (2016, 2017, 2018b,d,e, 2019). The data sets can be accessed via the University of Edinburgh data share repository here: http://dx.doi.org/10.7488/ds/2329.

## Ethics Statement

This study was carried out in accordance with the recommendations of the Linguistics & English Language Ethics Committee of the University of Edinburgh, with written informed consent from all subjects. All subjects gave written informed consent in accordance with the Declaration of Helsinki. The protocol was approved by the Linguistics & English Language Ethics Committee.

## Author Contributions

JH and CH contributed equally to the reported research and the writing of the article.

### Conflict of Interest

The authors declare that the research was conducted in the absence of any commercial or financial relationships that could be construed as a potential conflict of interest.

## Footnotes

^1^In German and Icelandic in particular there are exceptions to this generalization, as will be discussed later.

^2^It should also be noted that in the proposal of Bjorkman and Zeijlstra (2019) that argues for Agree to be uniformly “upward” (with the goal c-commanding the probe), the checking relation established by Agree is followed by a second step of valuation, and in certain circumstances this can have the result that agreement can obtain between a probe and a c-commanded element, for example between finite T and a lower nominative argument. This possibility only arises if there is a higher argument that is featurally defective in some way. Bjorkman and Zeijlstra do not discuss SCCs, but it seems that the most natural application of their proposal to account for the possibility of DP2 agreement would require that DP1 be analyzed as featurally deficient. Such an analysis has been advanced in Béjar and Kahnemuyipour (2017, 2018); see Hartmann and Heycock (2018a,c) for arguments against the claim of featural deficiency for DP1.

^3^For these cases where the “low” nominative argument is the subject of a non-finite clause, default (3rd person singular) agreement in the matrix is grammatical for most, possibly all speakers, while this type of default agreement is unacceptable when the nominative is a co-argument of the dative. See Sigurðsson and Holmberg (2008) for details, including interspeaker variation, and the further data and discussion in Thráinsson et al. (2015).

^4^As a referee points out, further evidence in the same direction is that the inclusion of the copula improves (12b), but not (12a):

^5^There are limited/sporadic exceptions to this generalization. Typically they involve cases where the initial nominal is ambiguous/underspecified for number, as with light-headed relatives like (i)a or cases that might involve NP ellipsis like (i)b: For some further discussion see Heycock (2012), p. 213.

^6^One exception to this is if DP1 can be a plurale tantum nominal, semantically singular but formally plural. Thus for example the Icelandic word *upptök* ‘cause(s)' is formally plural but semantically singular, and can appear as the first DP in SCCs like (i) Such cases, and the agreement patterns that are found there, are discussed in Hartmann and Heycock (2018a). A reviewer points out that for speakers of varieties of English where collective nouns like *council* are unambiguously singular, other exceptions would be examples like (ii):

^7^We take it that *It is I* to be a frozen form in Modern Day English, not part of the productive syntax.

^8^Clearly, for this kind of movement to be possible, it must be the case that this longer movement is not in violation of the PIC or any equivalent locality condition. There are already arguments in the literature that any impenetrability induced by v has to be modulated in some way to avoid constraints on locality that are empirically too strict; for example ruling out agreement with “low” nominatives in Icelandic of the type illustrated in (6) above. The proposal in Chomsky (2001) is to weaken the PIC so that the complement of a phase is only spelled out when the next phase head (C, in this case) is merged. An alternative, defended for example in Keine (2017) on the basis of evidence from Hindi-Urdu, is to reject the hypothesis that vP defines a phase at all. Note that also under an account along the lines of Béjar and Kahnemuyipour (2017), an agreement probe on T has to be able to reach a DP within the small clause in order to account for DP2 agreement.

^9^Note that Béjar and Kahnemuyipour (2018) have adjusted their approach to allow for DP1 to have accessible number features in the light of the results in Hartmann and Heycock (2018a); we will come back to why this is still not enough in section 5.3, see also Hartmann and Heycock (2018c).

^10^There are two reasons why direct comparison in one model is not easily possible: first, absolute ratings might differ because the reference sentences used for the different languages might not be completely equivalent in their acceptability, so that scales might differ between languages. Second, there are morphological differences between the languages which make it impossible to test all the same conditions for all four languages.

^11^This package was developed by E. Onea at the Göttingen Courant Research Centre “Text Structures” at Göttingen University, see https://onexp.textstrukturen.uni-goettingen.de.

^12^We indicate in the tables whether DP2 was a plural definite, or a plural pronoun. As mentioned in section 3.2, when DP1 and DP2 differ in number, it is always DP1 that has to be singular. This might lead to the conclusion that what appears to be DP1 agreement is actually “default” singular agreement. We address this issue in section 4.2

^13^Even in English there can be ambiguities in root clauses due to the other A′ predicate fronting construction illustrated in (18) above.

^14^We tested agreement with a 3pl DP2 in all four languages. Agreement with 1/2 person DP2 in root clauses was tested only in German, see below.

^15^A referee notes however that since the examples with and without negation were presented within the same experiment so that the presence/absence of negation could be treated as a variable, there could be an effect of structural priming from the negative sentences to the non-negative ones. To the extent that this kind of priming may have occurred, we cannot be certain that the non-negative sentences would be parsed in the same way in the absence of this hypothesized effect.

^16^Note that in this case we tested V2 clauses in German; embedded clauses for the other three languages.

^17^A referee suggests that the variability in agreement within the results for a single language might be due to speakers' varying in their interpretation of the sentences in the materials as SCCs or as other types of copular clause (predicational or equative). We have outlined in section 3.3 some ways in which we tried to eliminate or at least reduce this confound in our examples. We can now see from these results that the extent of variability is different in the different languages (for example, even if we consider only the data from root clauses in Table 1, German is much less variable than any of the other languages). As the items we used in our material are as similar as possible across the experiments in the different languages, this between-language difference in the extent of variation suggests that at most a small amount of this variation could be due to individual variation in construal.

^18^In this table we give the differences in z-scores for each language, rather than absolute values, in order to abstract away from other differences between the languages (see Hartmann and Heycock, 2018b for detailed discussion). So here the “advantage” of DP2 agreement is the figure that results from subtracting z-scores for DP1 agreement from z-scores for DP2 agreement.

^19^Note that we did not run a comparable experiment for differences with number mismatch in Icelandic. Observe also that in this judgment task in Icelandic, the ratings for DP2 agreement with a 2nd plural pronoun that contribute to the figure here were for examples showing full Number+Person agreement; this may be part or all of the reason why DP2 agreement here appears to be disfavoured in comparison with its frequency as shown in Table 2.

^20^Again, our point here is that variation exists, the distribution of this variation between and within speakers is the next step to take.

^21^So far, we only have acceptability ratings for Dutch and German on this phenomenon.

^22^In this tree structure we assume that (i) German has a TP and (ii) that DP1 in SCCs moves to Spec,TP, neither of which is crucial for our analysis. At the same time, both assumptions are under discussion: e.g., Haider (1997) and Sternefeld (2009) have argued against the presence of T in German. If T is absent, the agreement probe remains on the highest verbal projection. In both approaches, with or without T, the crucial aspect for our analysis is that DP1 moves directly above the agreement probe. Second, given that there is a TP in German, it has been extensively discussed whether subjects move into Spec,TP (see among others, Abraham, 1993; Haider, 1993; Alexiadou and Anagnostopoulou, 1998; Biberauer, 2004) or can just simply A-scramble to adjoin to TP. Again the two options are both compatible with our analysis as long as DP1 A-moves to a position above the agreement probe.

^23^In German, based on the data we obtained, there seems to be a small group of speakers who consistently prefer DP2 agreement in XP-initial clauses, however, there are many speakers whose ratings on both DP1 and DP2 agreement in this condition vary considerably.

^24^For evidence that the dative is indeed the subject in examples like these, see e.g.,Zaenen et al. (1985) and Sigurðsson (1989).

^25^In Hartmann and Heycock (2018d) we provide experimental data that supports the existence of an effect of syncretism in Dative–Nominative structures in Icelandic, and argue on that basis that the multiple agreement approach is the most plausible. The syncretism effect is however far from categorical, and we argue that syncretic forms are a repair mechanism which is not available to all speakers in the same degree.

^26^Please be aware that the data in the language specific summaries in Tables 7–10 are based on three/four different experiments: the data in each box bounded by lines on top/bottom and left/right belong together. There are differences in the ratings across experiments, and absolute z-score values cannot be compared across experiments. We do find differences between experiments, which may result from the fact that we had different participants for the different experiments within a single language and/or that we used different fillers in the respective experiments. We have to leave the work of teasing apart these possibilities to future research.

^27^Note: the person agreement production data reported here on German is from root clauses, whereas in the rating study we used embedded clauses. Note also that in the first two rows of all the following tables, which show the production and rating of different agreement options when DP1 and DP2 differ only in number, DP2 was a full (lexically headed) DP, while in the other rows, where the mismatch is for person (plus number in some cases), it was necessarily a pronoun for 1/2 person. For third person, we indicate in the tables whether or not we had a full (non-pronominal) DP or a pronoun. As mentioned in section 3.3, we were not able to make an entirely minimal comparison in German because the 3rd person plural pronoun is homophonous with 3rd singular feminine (*sie* in both cases) and so could not be used.

^28^In this paper, we argue that the crucial difference between the dative-nominative construction and the cases discussed here is that the dative is an intervener: it agrees with the probe but does not halt it. Thus the probe can enter a agree relationship with both the dative and the nominative, giving rise to syncretism effects. The nominatives in SCCs halt the probe, so there is no multiple agreement and no true syncretism effect.

^29^There was in fact one further condition, which was like the (b) condition except that DP1 matched the type used in the (d) condition (e.g., *winner* rather than *problem*), this was mentioned briefly in section 3.3 above. As the type of DP1 was shown not to have a significant effect, we do not discuss it further here; for detailed discussion see Hartmann and Heycock (2017, pp. 249–261).

^30^As DP1 in SCCs cannot be 1st or 2nd person, as discussed earlier, there is no way to test in this specific case whether or not this is DP1 agreement or default. However, as we have argued above, where we were able to test for “default” agreement in SCCs, it was always judged ungrammatical, so we exclude it as option here, too.

^31^Note that in Preminger (2011, 2014) the order of the two heads has number higher than person in contrast to the proposal in Sigurðsson and Holmberg (2008). As will be shown below, our data and analysis support the order proposed in Sigurðsson and Holmberg (2008), which is consistent with the morphology on the verb in Icelandic.

^32^It is also the case that in the rating study—but not the production study—the embedded clauses all contained negation. This makes the sentences more complex and is thus expected to depress the ratings to some extent.

^33^We had two more conditions including negation for condition A and B; as there was no difference with or without negation (as expected), we do not discuss this here any further.

^34^The figures in column 4 (ϕ-features on the copula matching DP1) are the number of 3rd singular verb forms; the figures in column 5 are the number of verb forms showing plural agreement (in A and C) and 2nd person singular agreement in B.

^35^The production data alone are consistent with an alternative analysis, namely that the lower proportion of apparent DP2 agreement when DP2 is 2nd person singular could be due to a morphological conflict that does not arise in the plural due to syncretism (an explanation given for the“person effect” in dative-nominative constructions in Icelandic, see section 2 above). However, this alternative can be ruled out by taking into consideration also the corresponding rating examples. We do not discuss this further here, see Hartmann and Heycock (2018e) for details.

^36^As noted in section 4.3 we have production data for this phenomenon from all four languages, but rating data only from Dutch and German.

^37^Some additional proposal would have to be made for this type of analysis to be extended to account for the agreement pattern in the XP–V_*fin*_–DP1–DP2 order discussed in section 4.3. Further, as just discussed, it is not clear that the number-only DP2 agreement pattern attested in Icelandic could be accounted for under this analysis.
